# Comparison of small n statistical tests of differential expression applied to microarrays

**DOI:** 10.1186/1471-2105-10-45

**Published:** 2009-02-03

**Authors:** Carl Murie, Owen Woody, Anna Y Lee, Robert Nadon

**Affiliations:** 1 McGill University and Genome Quebec Innovation Centre, 740 avenue du Docteur Penfield, Montreal, Quebec H3A 1A4, Canada; 2 Department of Human Genetics, McGill University, Montreal, 1205 avenue du Docteur Penfield N5/13, Quebec H3A 1A4, Canada

## Abstract

**Background:**

DNA microarrays provide data for genome wide patterns of expression between observation classes. Microarray studies often have small samples sizes, however, due to cost constraints or specimen availability. This can lead to poor random error estimates and inaccurate statistical tests of differential expression. We compare the performance of the standard t-test, fold change, and four small n statistical test methods designed to circumvent these problems. We report results of various normalization methods for empirical microarray data and of various random error models for simulated data.

**Results:**

Three Empirical Bayes methods (CyberT, BRB, and limma t-statistics) were the most effective statistical tests across simulated and both 2-colour cDNA and Affymetrix experimental data. The CyberT regularized t-statistic in particular was able to maintain expected false positive rates with simulated data showing high variances at low gene intensities, although at the cost of low true positive rates. The Local Pooled Error (LPE) test introduced a bias that lowered false positive rates below theoretically expected values and had lower power relative to the top performers. The standard two-sample t-test and fold change were also found to be sub-optimal for detecting differentially expressed genes. The generalized log transformation was shown to be beneficial in improving results with certain data sets, in particular high variance cDNA data.

**Conclusion:**

Pre-processing of data influences performance and the proper combination of pre-processing and statistical testing is necessary for obtaining the best results. All three Empirical Bayes methods assessed in our study are good choices for statistical tests for small n microarray studies for both Affymetrix and cDNA data. Choice of method for a particular study will depend on software and normalization preferences.

## Background

Microarrays provide large-scale comparative gene expression profiles between biological samples by detecting differential expression for thousands of genes in parallel. Typically, systematic error (bias) in the measurements is removed at the background correction and normalization steps, and is followed by statistical testing. In the most common type of study, statistical testing produces a list of genes that are differentially expressed across two or more classes (e.g., patient groups, treated vs. control animals, etc.) [1].

Extensive research has shown that choice of pre-processing methods designed to correct for bias in the measurements can have a substantial impact on rank ordering of gene expression fold-change (FC) estimates for both cDNA [2] and oligonucleotide data [3-5] and the AffyComp website [6]. One major disadvantage of FC estimates, however, is that they do not take the variance of the samples into account. This is especially problematic because variability in gene expression measurements is partially gene-specific [7], even after the variance has been stabilized by data transformation [8].

There is consensus in the statistical community that statistical tests of differential expression are preferred over FC for inference [9]. One advantage is that they standardize differential expression by dividing FC measurements by their associated standard error, rescaling FCs to a common metric. Moreover, associated output such as p values, effect sizes, and confidence intervals can be used for various purposes such as false positive control [10] and meta-analysis [11].

Although microarray studies with sample sizes of five or more observations per class are becoming increasingly common, cost considerations and the need for small scale initial studies mean that many studies are conducted with smaller sample sizes. The use of classical statistical tests such as the t-test is sub-optimal for small sample studies, however, because of low statistical power for detection of differential expression. This has led to the development of small sample size error estimation methods which borrow information from the entire data set or from a subset of the data to improve error estimation accuracy and precision [9,12].

There has been a number of studies analysing the performance of statistical tests applied to microarray data but few have used data where the differentially expressed genes are known in advance. Qin and Kerr [2] compared normalization methods and test statistics using data sets with six known differentially expressed genes and showed that the standard t-statistic performed worse than those that used variance estimates that incorporated information from all genes. Sioson et al. [13] compared statistical methodologies of two software applications using qRT-PCR of a subset of genes and Chen et al. [14] used a consensus of differentially expressed genes across statistical methods to analyze performance. None of these studies, however, evaluated statistical tests designed for small sample size experiments.

A number of studies have examined small n tests. Kooperberg et al. [15] compared the performances of various 2-group statistical tests with empirical and simulated data. Distributions of p-values were generated based on data from the same experimental group (and consequently it was known that there were no differences) or data from different experimental groups which were known to differ. The high performing tests generated p-value distributions consistent with a null distribution in the former case and produced the largest number of small p-values in the latter case. Using the limma method [16] as an exemplar, they concluded that an Empirical Bayes approach to statistical testing provided good power while accurately controlling false positive rates for small n microarray experiments. Cui and Churchill [17] reviewed a number of statistical tests for use with microarray data, including one small n test, but provided no comparative analysis. Tong and Wang [18] used simulated data to explore the theoretical properties of shrinkage methods of estimating variance and found that they outperformed tests that use only the sample variance. Hu and Wright [19] and Xie et al. [20] both compared a number of statistical tests, including several small n tests, using a consensus list of differentially expressed genes from all methods. Hu and Wright [19] found that, based on false discovery rates, tests that model the variance/intensity relationship and use variance estimates generated with information from all genes performed the best. Xie et al [20] showed that there was comparability of results for only a few of the methods but the Lönnstedt and Speed [21] B-statistic, which is monotonically equivalent to the limma [16] and BRB [22] t-statistics, had the lowest false positive rate. Jefferey et al. [23] looked at the use of statistical tests, including several small n tests, in feature selection for group classification; the results varied greatly across gene list and sample size but the Empirical Bayes t-statistic performed well across all sample sizes. Most of these studies compared methods using comparability of results, estimated false discovery, family wise error rates, or p-value distributions to assess performance. In this paper we focus on each method's ability to detect genes which have been spiked at different concentrations across samples (i.e., which are differentially expressed by design).

We use simulated data, two publicly available empirical Affymetrix Latin-Square data sets, and two cDNA data sets in which the differentially expressed genes are known to assess the relative performances of FC, t-statistic, and four small sample size statistical tests which all borrow strength from other genes in different ways. For consistency, we refer to the various tests by their software implementation. The Local Pooled Error method (LPE) z-statistic [24] is based on pooling errors for genes with similar intensities. The three Empirical Bayes methods were: the BRB t-statistic [22] which combines gene-specific error estimates with a common error estimate obtained from the distribution of the variances across all genes; the CyberT regularized t-statistic [25] which combines gene-specific error estimates with a local pooled error estimate based on genes with similar intensities; and the limma method [16] which combines a fitted linear model of gene expression data with a variance estimate into a moderated t-statistic. All methods gain degrees of freedom over the standard t-test and in theory should provide greater sensitivity with no loss in specificity.

## Data

### Latin Square spike-in data

The HGU133A and HGU95 Latin Square data sets [26] are based on a 14 × 14 Latin Square design of "spiked-in" transcripts (14 concentrations per microarray chip × 14 groups) with three replicates for each group. The concentrations for the "spiked-in" transcripts were doubled for each consecutive group (0 and 0.125 to 512 pM inclusive for HGU133A; 0 and 0.25 to 1024 pM inclusive for HGU95).

We compared performance of the statistical tests (see Methods) for detecting two-fold differential expression after the data had been normalized by six popular expression summary algorithms: MAS 5.0 [27], dChip [28], RMA [3], gcRMA [29], VSN [30], and LMGene [31]. Data were analysed on the log scale for the first four methods and on a generalized log (glog) scale for the latter two methods.

### cDNA spike-in data

Two spotted cDNA experiments [32] were also used to compare statistical test performance. The first experiment compared two aliquots from the same mouse liver RNA sample, and the second compared one RNA sample from mouse liver to a pooled sample of five different mouse tissues (liver, kidney, lung, brain, and spleen). In both experiments six transcripts were "spiked-in" at a three-fold difference in concentration between the two groups. All cDNA slides measured each "spike-in" transcript at 48 different well locations for a total of 288 "spikes" in each experiment.

The data were normalized using the lowess normalization of LMGene [31], with and without a glog transformation and background correction (foreground median intensity minus background median intensity), and the VSN method [30]. The LMGene lowess normalization method is equivalent to the intensity-dependent normalization used in Yang et al. [33]. MvA plots and their corresponding lowess fits for each cDNA array are shown in Additional file 1.

### Simulated data

Two limitations of the above spike-in data sets make them less challenging than most biologically motivated studies: variation across technical replicates is lower than that typically observed across biological replicates [34-36] and many biological effects of interest may be smaller than two-fold. Also, there is only a small number of "spiked-in" transcripts in each Latin Square experiment, which can lead to increased variability in the algorithm performance metrics [37]. Simulated data were generated to offset these limitations: Null hypothesis and "spike-in" data (in which a subset of the simulated genes were upwardly expressed in one group) were used to examine false positive rates (FPR), genes incorrectly labeled as differentially expressed, and true positive rates (TPR), genes correctly labeled as differentially expressed. The "spike-in"data incorporated varying degrees of variability between replicate measurements and genes were differentially expressed across a range of fold changes to offset the limitations of the Latin Square data sets.

Each simulation experiment consisted of 1000 iterations of ten thousand genes for each of two groups (control and treatment) with 3, 4, or 5 replicates per gene. Gene intensity values were generated by randomly selecting from a *N*(*u*, *σ*^2^) distribution, where *u *is the "true" expression intensity (drawn from a *N*(7, 1) distribution) for a single gene in log_2 _scale and *σ*^2 ^is the random error associated with the gene's expression measurement. In the null hypothesis data, each gene-specific *u *remains constant across both groups; in the "spike-in" data, *μ *was upwardly regulated by a specified fold change for 100 genes in the treatment group.

For each "spiked-in" gene, *μ *was assigned a value from a set of 10 log_2 _values ranging from 4 to 8.5 in 0.5 increments. The log_2 _fold change applied to the "spiked-in genes" was implemented by an additive adjustment to their *u *in the treatment group chosen from a range of 10 values from 0.2 to 1.1 (fold change from 1.15 to 2.15) in 0.1 increments. There are 10 "spiked-in" genes at each of the 10 concentration values and each of those 10 genes at a particular concentration value is upwardly regulated by one of the 10 different fold changes.

The random error of each gene is derived from one of three variance models: common, inverse gamma, and local (Table 1). In the common error model, the population variance (*c*) of the random error term (*ϵ*) is a constant across all genes. In the inverse gamma variance model, *c *is drawn randomly from an inverse gamma distribution, *IG*(3, *b*), where 3 is the shape parameter and b is the scale parameter. In the local variance model, *ϵ *is a combination of additive (*ϵ*_*a*_) and proportional (*ϵ*_*p*_) components, as outlined in Rocke and Durbin [38].

**Table 1:** Simulated data error models

**Variance Model**	**Expression Intensity (*x*_*i*_*_j_*)**	**Random Error**	**Parameters**
Common	*μ*_*i *_+ *ϵ*_*i*_	*ϵ*_*i *_~ *N *(0, *c*)	*c *∈ (.1, .05, .01)

Inverse Gamma	*μ*_*i *_+ *ϵ*_*i*_	*ϵ*_*i *_~ *N *(0, *IG*(3, *b*))	*b *∈ (.2, .1, .05)

Local	*log*_2_($\mu_{i}e^{\epsilon_{pi}}$ + *ϵ*_*ai*_)	*ϵ*_*ai *_~ *N *(0, *c*) *ϵ*_*pi *_~ *N *(0, .1)	*c *∈ (50, 25, 5)

The Expression Intensity column shows how the replicate intensity values were generated for each transcript. The Random Error column shows the method of calculating specific *ϵ *values. The Parameters column shows the values that were used to generate high, medium, and low variances, respectively.

**Figure 1:**
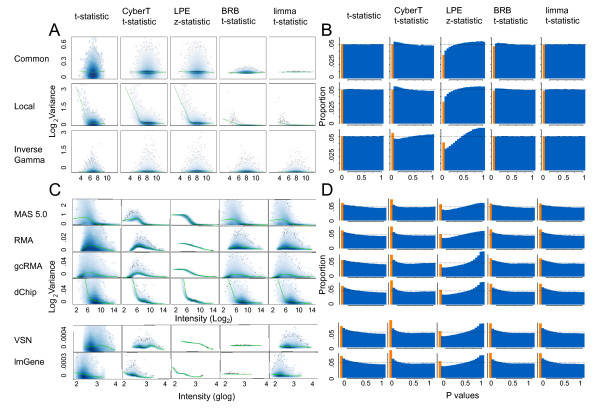
**Scatter plots of intensity vs log variance and histograms of p values for all methods and data sets**. A and B show simulated high variance null hypothesis data (*n *= 6); C and D show HGU133A data with the "spiked-in" genes removed. (A) Scatter plot of intensity versus pooled log variance (1 iteration). (B) Histograms of p values (1000 iterations). (C) Scatter plot of intensity versus log pooled variance. (D) Histograms of p values (average of all 14 comparisons). Orange columns in histograms correspond to the proportion of p values ≤ 0.05.

The parameters of each variance model were varied to create data sets from low variance (approximating the Latin Square and some cell line data sets) to medium and high variance (approximating the increased variance observed in experimental animal and patient tissue samples [34,39]. Scatter plots of expression intensity versus log pooled variance of the three variance models with high variance are shown in Figure 1A.

## Methods

See Additional file 2 for software versions and availability.

### Fold-change

The Fold-Change (FC) ratio is typically represented on the log_2 _scale:

(1) $$FC={\bar{x}}_{1}-{\bar{x}}_{2}$$

where ${\bar{x}}_{1}$ and ${\bar{x}}_{2}$ are the means of the two groups' logged expression values.

### Independent t-test

The independent t-statistic provides a standardized estimate of differential expression according to the following formula:

(2) $$t=\frac{1}{\sqrt{\frac{1}{n_{1}}+\frac{1}{n_{2}}}}\frac{{\bar{x}}_{1}-{\bar{x}}_{2}}{{\hat{\sigma }}_{pool}}$$

where

(3) $${\hat{\sigma }}_{pool}^{2}=\frac{(n_{1}-1){\hat{\sigma }}_{1}^{2}+(n_{2}-1){\hat{\sigma }}_{2}^{2}}{n_{1}+n_{2}-2}$$

${\hat{\sigma }}_{i}^{2}$ and *n*_*i *_are the variance and number of replicates for the *i*th group, respectively. ${\hat{\sigma }}_{pool}^{2}$ is the weighted average of the two groups gene-specific variance. The associated probability under the null hypothesis is calculated by reference to the t distribution with n_1 _+ n_2 _- 2 degrees of freedom.

### Local pooled error (LPE) z-statistic

Jain et al.'s (2003) local pooled error test is based on the model that genes with similar observed intensities have similar true variances and is calculated as follows:

(4) $$z=\frac{Median_{1}-Median_{2}}{\sigma_{pool}}$$

where

(5) $$\sigma_{pool}^{2}=\frac{\pi }{2}(\frac{\sigma_{1}^{2}}{n_{1}}+\frac{\sigma_{2}^{2}}{n_{2}})$$

The fold change is calculated using a difference of medians and the gene specific $\sigma_{i}^{2}$ is obtained from a calibration curve derived from pooling the variance estimates of each gene's replicate expression measures with the variance estimates of other genes with similar expression values. *n*_1 _and *n*_2 _are the sample sizes for the two groups. The variance is adjusted by *π*/2 due to the increased variability of using medians when calculating the fold change. The associated probability of the z-statistic under the null hypothesis is calculated by reference to the standard normal distribution.

### Statistical tests with Empirical Bayes variance estimators

The following three tests use an Empirical Bayes method to estimate error associated with differential expression and use a statistical test identical in form to the independent t-statistic shown in equation 2. The tests use a posterior variance, ${\tilde{\sigma }}^{2}$, in place of the pooled variance, $\sigma_{pool}^{2}$, in the t-test. Using Bayes rule, the posterior variance, ${\tilde{\sigma }}^{2}$, for each gene becomes a combination of the observed gene-specific error and an estimate obtained from the prior distribution according to the following formula.

(6) $${\tilde{\sigma }}^{2}=\frac{(d_{g}){\hat{\sigma }}_{g}^{2}+(d_{0})\sigma_{0}^{2}}{d_{g}+d_{0}}$$

*d*_*g *_are the degrees of freedom and ${\hat{\sigma }}_{g}^{2}$ are the gene specific variances obtained from the experimental data. *d*_0 _and *σ*^2 ^are the prior degrees of freedom and variance estimates respectively [16]. The three methods differ in how they estimate the parameters *d*_0 _and $\sigma_{0}^{2}$.

#### Limma t-statistic

Smyth [16] developed the hierarchical model of Lönnstedt and Speed [21] into a general linear model that is more easily applied to microarray data. It is implemented using the R statistical language in the limma bioconductor package [40]. This method is based on a model where the variances of the residuals vary from gene to gene and are assumed to be drawn from a chi-square distribution.

The linear model is as follows:

(7) *E*(*y*_*g*_) = *Xα*_*g*_

where *y*_*g *_is the expression summary values for each gene across all arrays, *X *is a design matrix of full column rank and *α*_*g *_is the coefficient vector. The contrasts of coefficients that are of interest for a particular gene *g *are defined as *β*_*g *_= *C*^*T*^*α*_*g*_. Although this approach is able to analyse any number of contrasts, we examine two sample comparisons only so *β*_*g *_can be defined as the log fold change ${\hat{x}}_{1}-{\hat{x}}_{2}$ (Equation 1).

The contrast estimators, *β*_*g*_, are assumed to be normal and the residual variances, $s_{g}^{2}$, are assumed to follow a scaled chi-square distribution. Under this hierarchical model the posterior variance, ${\tilde{s}}_{g}^{2}$, takes the form:

(8) $${\tilde{s}}_{g}^{2}=\frac{d_{0}s_{0}^{2}+d_{g}s_{g}^{2}}{d_{0}+d_{g}}$$

where *d*_0 _and $s_{0}^{2}$ are the prior degrees of freedom and variance and *d*_*g *_and $s_{g}^{2}$ are the experimental degrees of freedom and the sample variance for a particular gene *g*, respectively. Because we examine only two sample comparisons, *d*_*g *_will always be equal to *n *- 2 where *n *is the total number of replicates. The two prior parameters, *d*_0 _and $s_{0}^{2}$, can be interpreted as the degrees of freedom and variance of the prior distribution respectively. The prior parameters are estimated by fitting the logged sample variances to a scaled F distribution.

The moderated t-statistic is defined by:

(9) $$\tilde{t}=\frac{1}{\sqrt{\frac{1}{n_{1}}+\frac{1}{n_{2}}}}\frac{\hat{\beta }}{{\tilde{s}}_{g}}$$

The associated probability of the moderated t-statistic for the two sample case under the null hypothesis is calculated by reference to the t-distribution with *d*_0 _+ *d*_*g *_degrees of freedom.

#### BRB t-statistic

Like the limma model, Wright and Simon's [22] RVM t-statistic, labelled BRB t-statistic after the BRB software, assumes a random variance model where the variances of the residuals vary from gene to gene and are random selections from a single prior inverse gamma distribution, which is a more general form of the chi-square. The two models differ, however, in the method of the prior parameter estimations. In the BRB model, the estimate of the gene specific error corresponds to the sample variance of the gene's replicate expression measures; the prior estimate corresponds to the mean of the inverse gamma prior distribution fitted to the sample variances. The prior distribution is *P*(*μ*|*σ*^2^)*P*(*σ*^2^), where the marginal *P*(*σ*^2^) is the scaled inverse gamma and the conditional distribution *P*(*μ*|*σ*^2^) is normal. That is, the random error associated with each gene's measurement is assumed to be distributed normally with a mean of zero and the variance of the intensity distribution is assumed to be randomly drawn from a prior inverse gamma distribution whose parameters need to be estimated. The posterior variance for each gene is a weighted average of its observed sample variance and the mean of the prior variance distribution. The shape (*a*) and scale (*b*) parameters for the prior are estimated by fitting the entire collection of observed variances to an inverse gamma distribution. Rocke [41] has discussed a method of moments approach to estimate the parameters while Wright and Simon [22] have advocated a maximum likelihood approach to fit ab * ${\hat{\sigma }}^{2}$ to an *F*(*n *- *k*, 2*a*) distribution, where *n *is the total number of replicates and *k *is the number of groups. The maximum likelihood approach was found to have lower variability (data not shown) and was the method used in this study.

The BRB t-statistic for a particular gene takes the form:

(10) $$\tilde{t}=\frac{1}{\sqrt{\frac{1}{n_{1}}+\frac{1}{n_{2}}}}\frac{{\bar{x}}_{1}-{\bar{x}}_{2}}{\tilde{\sigma }}$$

where the posterior variance is

(11) $${\tilde{\sigma }}^{2}=\frac{(n-2){\hat{\sigma }}^{2}+2a{\left(ab\right)}^{-1}}{n-2+2a}$$

${\hat{\sigma }}^{2}$ is the gene specific sample variance with degrees of freedom of *n *- 2. The prior variance estimate((*ab*)^-1^) is the mean of the fitted inverse gamma distribution with degrees of freedom of 2*a*. The limma prior parameters *d*_0 _and $s_{0}^{2}$ are equivalent to the BRB prior parameters 2*a *and (*ab*)^-1^. A large number of replicates will give increased weight to the observed variance while a high value for *a *gives increased weight to the prior variance. A large *a *means that the inverse gamma is highly peaked making it more likely that the true variance for any particular gene is close to (*ab*)^-1^. In the two-sample case, the associated probability of the t-statistic under the null hypothesis is calculated by reference to the t-distribution with *n *- 2 + 2*a *degrees of freedom.

#### CyberT t-statistic

The Baldi and Long [25] regularized t-statistic, labelled the CyberT statistic after the Cyber-T software, combines elements of both the LPE and the two previous Empirical Bayes methods. Like LPE, variance estimates are obtained by pooling across genes with similar intensities.

A gene specific running average of the variance is used for the prior variance estimate. The running average variance is calculated by averaging the variance of a particular gene and the variances of the *z *number of genes on either side of that gene in the ordered distribution. The total window size (*w*) for the running average is equal to 2*z *+ 1. Accordingly, for pair, (*x*_*i*_, $\sigma_{i}^{2}$), ordered by *x*_*i*_, the running average is generated using:

(12) $$\sigma_{0i}^{2}=\frac{\sigma_{i-z}^{2}+\ldots +\sigma_{i}^{2}+\ldots +\sigma_{i+z}^{2}}{w}$$

The degrees of freedom (*v*_0_) for the running average is a user supplied theoretical value based on the belief in the quality of the running average variance. The heuristic suggested by Baldi and Long [25] of using three times the number of replicates was used for the running average degrees of freedom in the present study.

The CyberT t-statistic takes the form:

(13) $$\tilde{t}=\frac{1}{\sqrt{\frac{1}{n_{1}}+\frac{1}{n_{2}}}}\frac{{\bar{x}}_{1}-{\bar{x}}_{2}}{\tilde{\sigma }}$$

where the posterior variance is

(14) $${\tilde{\sigma }}^{2}=\frac{(n-2){\hat{\sigma }}^{2}+v_{0}\sigma_{0}^{2}}{n-2+v_{0}}$$

${\bar{x}}_{1}$ and ${\bar{x}}_{2}$ are the sample means of the two groups. ${\hat{\sigma }}^{2}$ is the gene specific sample variance, *n *is the total number of replicates (*n*_1 _+ *n*_2_) per gene across treatment classes, *v*_*o *_is the degrees of freedom of the prior, and $\sigma_{0}^{2}$ is the gene specific running average variance. The associated probability of the t-statistic under the null hypothesis is calculated by reference to the t-distribution with *n *- 2 + *v*_0 _degrees of freedom.

## Results and discussion

### Descriptive comparisons

Figures 1A and 1C show the scatter plots of log pooled variance estimates (Equations 3, 5, 8, 11, and 14) versus average intensity across replicates of both groups for simulated and Affymetrix data, respectively. The t-statistic shows the unadjusted variance values before the small *n *methods are applied and can be used as a baseline for comparison. The scatter plots for the complete set of Latin Square comparisons and the null hypothesis data with low and medium variances are comparable to these plots (data not shown). With two exceptions (both with MAS 5.0) the four small *n *statistical tests reduced the variability of the pooled variance estimates relative to the unadjusted estimates of the t-test for all data sets; the range of points on the y-axis in Figures 1A and 1C is diminished along the smoothing curve. LPE in particular compressed the variance because the gene-specific variance estimates for each group are generated from a calibration function, which maps a single associated variance value per group to each intensity value. The pooled variance estimates of the BRB and limma method with the Latin Square data normalized by MAS 5.0 showed little compression. The degrees of freedom (*n *- 2 = 4) assigned to the gene-specific variance is much greater than the degrees of freedom (2*a *and *d*_0_) assigned to the prior variance estimate (1.2 and 1.4 for BRB and limma, respectively). Consequently the adjustment applied by the BRB method is negligible. In contrast, the degrees of freedom values for the data shown in Figure 1 for RMA, gcRMA, dChip, VSN, and LMGene were 6.8, 2.6, 3.8, 256, and 218 for BRB and 5.3, 2.6, 3.3, 5.8, and 9.16 for limma, respectively.

The BRB and limma methods produced similar results for all expression summary methods with the exception of the Affymetrix data normalized with VSN and LMGene which use a generalized log transformation. Although the BRB and limma methods generated similar prior variance parameters, the former generated much higher prior degrees of freedom than the latter. The BRB prior degrees of freedom were on average 17 times higher than the limma degrees of freedom (236 to 14) for the VSN expression summary method and 38 times higher (278 to 7.5) for the LMGene expression summary method. For these data, the degrees of freedom for the sample variance were 4 so the posterior variance of the BRB method, which is a weighted average of the prior and sample variance, deviates only slightly from the prior variance across gene specific intensity (Figure 1C). The LMGene and VSN normalization methods both produced a narrow peaked sample variance distribution consisting of very small variances which will cause the BRB prior degrees of freedom parameter to be excessively large. The limma prior degrees of freedom parameter has a more appropriate value because it is calculated using the log of the sample variances which do not show such a narrow distribution [see Additional file 3]. The BRB t-statistic may not be appropriate for LMGene and VSN normalized data due to this issue.

Figures 1B and 1D show histograms of the p value distributions of the simulated and HGU133A data, respectively. Under the null hypothesis, the distribution of the p values should follow a uniform distribution. Deviation from a uniform distribution indicates lack of correspondence between the data and the assumptions of the statistical model. The histograms for the complete set of Latin Square experiments and the null hypothesis data with low and medium variances are comparable to these plots (data not shown).

The p value distributions for the t-statistic, BRB t-statistic and the limma moderated t-statistic followed the theoretically expected uniform distribution for all simulation models; the CyberT t-statistic deviated slightly from uniformity (Figure 1B). For the HGU133A data, all expression summary methods and statistical tests, with the exception of LPE, showed a rightward skew in their p value distributions and a greater than expected number of small p values (Figure 1D). Unlike the other statistical tests, the p value distribution for the LPE z-statistic deviated substantially from uniformity for all data sets and expression summary methods.

### Performance

A standard method of comparing the performance of different statistical methods is to use a partial AUC of an ROC graph calculated using only the lower end of the ordered distributions of p values. The pAUC has a value between 0 (worst performance) and 1 (perfect performance). These metrics were calculated for each of the 1000 iterations of the simulated "spike-in" data sets and for the 14 comparisons of each of the two Latin Square data sets. A particular false positive rate that applies commonly across the different distributions is often chosen. This has the advantage of ensuring that the same number of data points are used in the comparison but has the disadvantage that it ignores the actual p values of the data points being compared. An alternative is to use a cutoff based on a specific p value rather than using only the rankings which is more in line with how the test results would be used in applied contexts. We present results for the latter below and for the former in Additional file 4.

The average pAUC scores and rankings for all methods and data sets are shown in Table 2. The average true and false positive rates are shown in Tables 3 and 4 respectively.

**Table 2:** pAUC scores

		**t-stat**	**CyberT**	**LPE**	**BRB**	**limma**	**FC**	median pAUC
HGU133A	MAS 5.0	5 (.68)	1 (.75)	4 (.69)	2.5 (.70)	2.5 (.70)	6 (.18)	.70
	RMA	6 (.74)	1.5 (.85)	4.5 (.83)	1.5 (.85)	4.5 (.83)	1 (.84)	.84
	gcRMA	6 (.74)	1.5 (.85)	3 (.83)	4.5 (.79)	4.5 (.79)	1.5 (.85)	.81
	dChip	4 (.80)	2 (.83)	5 (.78)	2 (.83)	2 (.83)	6 (.76)	.82
	LMGene	6 (.75)	2.5 (.85)	2.5 (.85)	1 (.88)	4.5 (.84)	4.5 (.84)	.85
	VSN	6 (.74)	2.5 (.84)	5 (.82)	1 (.86)	2.5 (.84)	4 (.83)	.84

	avg. rank	5.5	1.8	4	2.1	2.4	4.2	

HGU95	MAS 5.0	3 (.59)	1 (.73)	2 (.63)	4.5 (.44)	4.5 (.44)	6 (.10)	.52
	RMA	6 (.68)	1 (.85)	1.5 (.73)	2.5 (.84)	2.5 (.84)	4 (.83)	.84
	gcRMA	6 (.76)	1.5 (.91)	5 (.81)	3.5 (.88)	3.5 (.88)	1.5 (.91)	.88
	dChip	5 (.73)	3 (.82)	6 (.52)	1.5 (.83)	1.5 (.83)	4 (.80)	.81
	LMGene	5.5 (.74)	1 (.86)	5.5 (.74)	4 (.84)	2.5 (.85)	2.5 (.85)	.85
	VSN	5.5 (.57)	4 (.76)	5.5 (.57)	2.5 (.80)	2.5 (.80)	1 (.82)	.78

	avg. rank	5.2	1.9	4.8	3.1	2.8	3.2	

cDNA liver vs liver	Loess	5 (.78)	4 (.88)	6 (.65)	2.5 (.96)	2.5 (.96)	1 (.97)	.92
	Loess(BC)	3.5 (.85)	1 (.96)	5 (.80)	3.5 (.85)	2 (.86)	6 (.66)	.85
	glog Loess	5 (.79)	2 (.96)	6 (.62)	3.5 (.95)	3.5 (.95)	1 (.98)	.95
	glog Loess(BC)	5 (.81)	1 (.96)	6 (.75)	4 (.93)	3.5 (.93)	3.5 (.94)	.93
	VSN	5 (.77)	3 (.96)	6 (.61)	3 (.96)	3 (.96)	1 (.97)	.96

	avg. rank	4.7	2.2	5.8	3.2	2.9	2.2	

cDNA liver vs pool	Loess	4 (.29)	3 (.39)	6 (.16)	1 (.44)	2 (.43)	5 (.17)	.51
	Loess(BC)	2.5 (.09)	2.5 (.09)	5 (.05)	2.5 (.09)	2.5 (.09)	6 (.02)	.09
	glog Loess	4 (.62))	1 (.73)	5 (.56)	2.5 (.69)	2.5 (.69)	6 (.08)	.65
	glog Loess(BC)	4 (.62)	1 (.73)	5 (.58)	3 (.65)	2 (.66)	6 (.06)	.64
	VSN	4 (.50)	1 (.57)	5 (.38)	2.5 (.55)	2.5 (.55)	6 (.05)	.53

	avg. rank	3.7	1.7	5.2	2.3	2.3	5.8	

Simulated	Common	6 (.32)	4 (.53)	5 (.42)	2 (.54)	2 (.54)	2 (.54)	.54
	Local	6 (.15)	4 (.27)	5 (.16)	2 (.60)	1 (.61)	3 (.31)	.29
	Inverse Gamma	5 (.39)	2 (.53)	6 (.35)	2 (.53)	2 (.53)	4 (.48)	.51

	avg. rank	5.7	3.3	5.3	2.2	1.2	3.3	

	total avg. rank	5.0	2.2	5.0	2.6	2.3	3.7	

Rankings from best to worst (1 to 5) of average pAUC scores (using a threshold of 0.05) of the six tests of differential expression. The average pAUC for each score is in parentheses. The LMGene package was used to apply the Loess normalization and glog transformation to the cDNA data. BC indicates that background correction was applied to the data before normalization.

**Table 3:** True positive rates

		**t-stat**	**CyberT**	**LPE**	**BRB**	**limma**	**FC**	median TPR
HGU133A 42 spikes	MAS 5.0	4.5 (.76)	1 (.80)	4.5 (.76)	2.5 (.78)	2.5 (.78)	6 (.30)	.77
	RMA	6 (.80)	2.5 (.87)	4.5 (.86)	2.5 (.87)	4.5 (.86)	1 (.89)	.87
	gcRMA	6 (.78)	2 (.88)	3 (.86)	4.5 (.83)	4.5 (.83)	1.5 (.89)	.85
	dChip	4 (.85)	2.5 (.87)	6 (.83)	1 (.88)	2.5 (.87)	5 (.84)	.86
	LMGene	6 (.80)	3.5 (.89)	3.5 (.89)	1 (.91)	5 (.88)	2 (.90)	.87
	VSN	6 (.79)	3 (.87)	5 (.85)	1 (.89)	3 (.87)	3 (.87)	.89

	avg. rank	6	2.5	4.5	1.8	3.4	2.5	

HGU95 12 spikes	MAS 5.0	2 (.73)	1 (.80)	3 (.71)	4.5 (.68)	4.5 (.68)	6 (.11)	.70
	RMA	5 (.82)	2.5 (.88)	6 (.81)	2.5 (.88)	2.5 (.88)	2.5 (.88)	.88
	gcRMA	6 (.85)	2 (.95)	5 (.89)	4 (.91)	3 (.92)	1 (.96)	.92
	dChip	5 (.84)	4 (.87)	6 (.66)	2 (.89)	2 (.89)	2 (.89)	.88
	LMGene	5.5 (.89)	2.5 (.85)	5.5 (.89)	2.5 (.89)	4 (.88)	1 (.90)	.89
	VSN	5 (.73)	4 (.82)	6 (.66)	2 (.85)	3 (.84)	1 (.86)	.83

	avg. rank	4.8	2.7	5.3	2.9	3.2	2.3	

cDNA liver vs liver 288 spikes	Loess	5 (.93)	4 (.95)	6 (.65)	2.5 (.97)	2.5 (.97)	1 (.98)	.96
	Loess(BC)	5 (.95)	2 (.98)	6 (.88)	2 (.98)	2 (.98)	4 (.97)	.98
	glog Loess	5 (.88)	3 (.97)	6 (.78)	3 (.97)	3 (.97)	1 (.98)	.97
	glog Loess(BC)	5 (.93)	3 (.97)	6 (.91)	3 (.97)	3 (.97)	1 (.98)	.97
	VSN	5 (.89)	2.5 (.98)	6 (.70)	2.5 (.98)	2.5 (.98)	2.5 (.98)	.98

	avg. rank	5.0	2.9	6	2.6	2.6	1.9	

cDNA liver vs pool 288 spikes	Loess	4 (.63)	1 (.84)	6 (.39)	2 (.83)	3 (.82)	5 (.48)	.73
	Loess(BC)	2.5 (.17)	1 (.21)	5 (.12)	4 (.16)	2.5 (.17)	6 (.05)	.17
	glog Loess	5 (.92))	1 (.98)	4 (.96)	2.5 (.97)	2.5 (.97)	6 (.18)	.97
	glog Loess(BC)	5 (.93)	1 (.98)	2 (.97)	3.5 (.96)	3.5 (.96)	6 (.16)	.96
	VSN	4 (.83)	1 (.97)	5 (.67)	2.5 (.92)	2.5 (.92)	6 (.27)	.88

	avg. rank	4.1	1.0	4.4	2.9	2.8	5.8	

Simulated 100 spikes	Common	6 (.48)	2.5 (.63)	5 (.51)	2.5 (.63)	2.5 (.63)	2.5 (.63)	.63
	Local	5 (.26)	4 (.37)	6 (.24)	2 (.69)	1 (.70)	3 (.42)	.40
	Inverse Gamma	5 (.54)	2 (.65)	6 (.51)	2 (.65)	2 (.65)	2 (.62)	.64

	avg. rank	5.1	2.5	5.1	2.5	2.6	3.2	

	total avg. rank	5.0	2.3	5.1	2.5	2.9	3.1	

Rankings from best to worst (1 to 5) of average true positive rate (TPR) scores of the six tests of differential expression. The average TPR for each score is in parentheses. The LMGene package was used to apply the Loess normalization and glog transformation to the cDNA data. BC indicates that background correction was applied to the data before normalization.

**Table 4:** False positive rates

		**t-stat**	**CyberT**	**LPE**	**BRB**	**limma**	**FC**
HGU133A 22258 genes	MAS 5.0	.064	.078	.060	.063	.062	.05
	RMA	.069	.084	.044	.078	.078	.05
	gcRMA	.050	.079	.031	.060	.061	.05
	dChip	.093	.115	.081	.105	.105	.05
	LMGene	.082	.099	.065	.095	.093	.05
	VSN	.074	.091	.049	.091	.088	.05

							

HGU95 12614 genes	MAS 5.0	.044	.05	.042	.046	.046	.05
	RMA	.040	.037	.011	.035	.034	.05
	gcRMA	.009	.018	.003	.017	.018	.05
	dChip	.043	.041	.003	.040	.040	.05
	LMGene	.040	.035	.008	.035	.033	.05
	VSN	.040	.035	.008	.035	.033	.05

							

cDNA liver vs liver 27648 genes	Loess	.002	.001	0	.001	.001	.05
	Loess(BC)	.01	.002	.001	.007	.007	.05
	glog Loess	.007	.002	0	.002	.002	.05
	glog Loess(BC)	.014	.008	.001	.011	.012	.05
	VSN	.006	.001	0	.001	.001	.05

							

cDNA liver vs pool 27648 genes	Loess	.138	.135	.034	.151	.151	.05
	Loess(BC)	.174	.171	.106	.169	.173	.05
	glog Loess	.233	.238	.151	.251	.252	.05
	glog Loess(BC)	.233	.235	.158	.238	.240	.05
	VSN	.244	.249	.183	.251	.252	.05

							

Simulated 9900 genes	Common	.048	.048	.032	.046	.047	.05
	Local	.044	.044	.028	.046	.045	.05
	Inverse Gamma	.048	.053	.039	.048	.048	.05

Average false positive rates (using a p value threshold of .05) of the six tests of differential expression. The LMGene package was used to apply the Loess normalization and glog transformation to the cDNA data. BC indicates that background correction was applied to the data before normalization.

**Figure 2:**
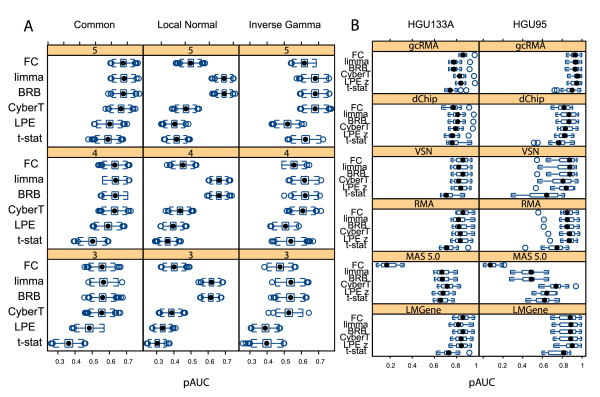
**Trellis boxplots of pAUC scores**. Trellis boxplots of pAUC using a *p *< 0.05 threshold. (A) Simulated "spike-in" variance models with 3, 4 and 5 replicates. (B) Latin Square data: processed with 6 expression summary methods. Differential expression tests: FC: Fold Change, limma: limma moderated t-statistic, BRB: BRB t-statistic, CyberT: CyberT t-statistic, LPE: LPE z-statistic, t-stat: t-statistic.

Figure 2 shows the boxplots of the pAUC of the statistical tests and FC for the "spike-in" high variance simulated data and for the two Latin Square data sets. For the simulated data, the CyberT, BRB, and limma tests consistently outperformed both the LPE and t-test methods. The pattern of results for the low and medium variance simulated data was the same as for the high variance simulated data, although, as expected, the effects were less pronounced [see Additional file 5]. All statistical tests performed similarly well for both Affymetrix Latin Square data sets, lending support to our argument that technically replicated data sets do not provide sufficient challenges for rigorous algorithm comparisons.

The three Empirical Bayes variance estimation methods, CyberT, BRB, and limma, performed comparably to FC with almost all low variance data sets (Table 2). They appreciably outperformed FC, however, with the liver versus pooled cDNA data set, which is characterized by higher variance than the other data sets due to the use of mRNA from multiple tissues, and MAS 5.0 normalized Latin Square data, which also has higher variability than the other normalization methods (Figures 1A and 1C). Greater variability randomly introduces a higher number of large fold changes between groups simply because of the greater range of expression intensities across replicate values. FC, which ignores variability, is less able to separate the higher number of random effects from the "spiked-in" effects than those methods that take the variability into account. For these latter data, the Empirical Bayes variance estimation tests of the loess/glog transformed data performed especially well (with or without background correction). These results show the limitations of using FC with highly variable data [36,42].

Based on average ranks, the CyberT t-statistic, BRB t-statistic, and the limma moderated t-statistic performed best across all data sets (Table 2). The top performers with the Latin Square data were able to find almost all of the differentially expressed genes and had comparable pAUC scores. The three Empirical Bayes variance estimation tests were in the top four performers with the low variability liver3 versus liver5 cDNA data and the top three with the higher variability liver versus pooled cDNA data. For the simulated data, the limma t-statistic performed best with the local variance model, tied with the CyberT t-statistic and the BRB t-statistic for the inverse gamma variance model, and tied with FC and the BRB t-statistic for best performance for the common variance model. The t-statistic and LPE z-statistic were the worst performers overall.

These results are similar to those generated by the pAUCs with the same number of data points across tests [see Additional file 4]. In both cases, the average performances of the three Empirical Bayes tests were ranked in the top three, FC was ranked fourth, and the LPE and t-test were ranked last [see Additional file 6].

**Figure 3:**
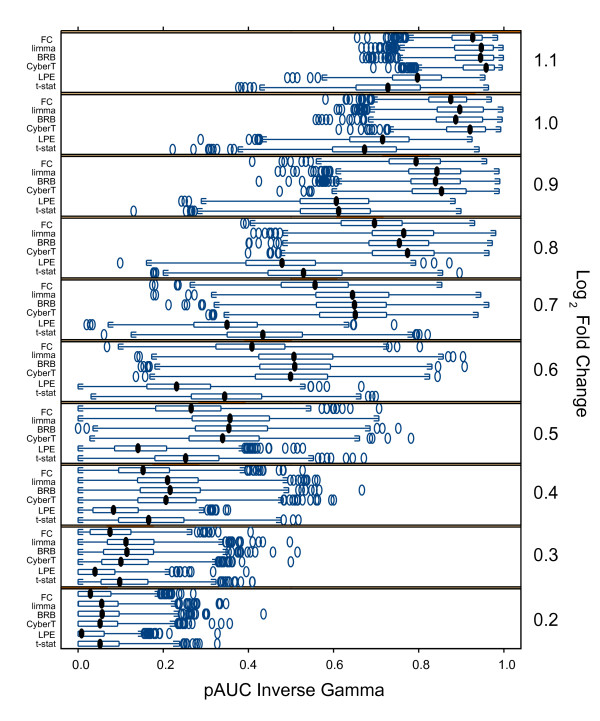
**Trellis boxplots pAUC scores by fold change for inverse gamma error model**. Inverse Gamma variance model simulated "spike-in" data: Trellis boxplots of pAUC using a *p *< 0.05 threshold. Results are shown for all "spike-in" log_2 _fold changes (0.2–1.1) separately. Differential expression tests: FC: Fold Change, limma: limma moderated t-statistic, BRB: BRB t-statistic, CyberT: CyberT t-statistic, LPE: LPE z-statistic, t-stat: t-statistic.

Figure 3 shows the pAUC of the methods for each of the 10 different fold changes of the spikes with the inverse gamma simulated data across the 1000 simulation iterations. There are ten "spiked-in" genes at different concentrations at each of the 10 different fold changes. The FC, CyberT, BRB, and limma methods did comparably well at high fold changes and comparably poorly at low fold changes. The FC method performed poorly relative to the three Empirical Bayes tests, however, within the middle FC range (*log*_2 _0.4–0.9). Moreover, the LPE's performance was worse than the other methods at all but the two highest FCs, for which the t-test performed worst.

With the common variance model, the high fold change transcripts were the most easily identified by all methods and it was within the medium fold change range that there was the best differentiation between the methods; this was also seen with lesser effect with the local variance model (data not shown).

### Concentration effects

We examined the data in greater detail by analyzing the true positive rates (TPR) and the false positive rates (FPR) separately, conditioned on spike-in concentration and using a *p *< 0.05 threshold.

**Figure 4:**
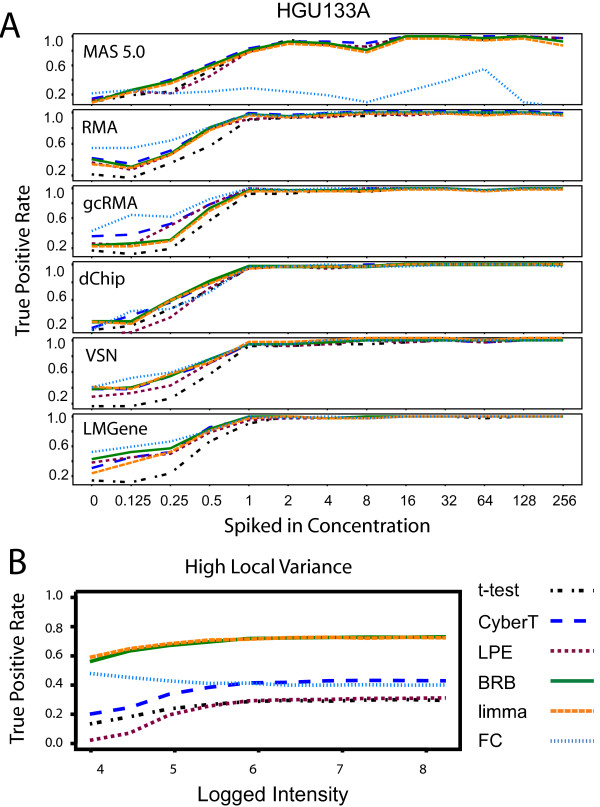
**True positive rates across concentration**. True positive rates across concentration using a *p *< 0.05 threshold. (A) HGU133A Latin Square data. (B) Local variance model "spike-in" simulated data with high variance (*n *= 3). Differential expression tests: FC: Fold Change, limma: limma moderated t-statistic, BRB: BRB t-statistic, CyberT: CyberT t-statistic, LPE: LPE z-statistic, t-stat: t-statistic. The limma and BRB t-statistic produce comparable results and consequently limma's orange line often conceals BRB's green line.

The differential expression tests found most of the Latin Square "spiked-in" genes at the mid to high concentrations with the HGU133A data (Figure 4A). There was, however, a drop in the TPR at low concentrations found across all combinations of expression summary methods and differential expression tests. FC had a very low TPR across all concentrations with the MAS 5.0 data. The CyberT, BRB, and limma tests performed as well or better than the LPE and t-test methods in limiting this effect. For the simulated local variance model, the BRB t-statistic and limma moderated t-statistic yielded substantially higher TPR values across all concentration levels, although there was a decrease in the TPR among low concentrations for all statistical tests (Figure 4B). For the common variance and inverse gamma simulated models, the BRB t-statistic, the CyberT t-statistic, the limma moderated t-statistic, and FC yielded similarly high TPRs across all concentrations; the t-test and the LPE z-test yielded lower TPR values across all concentrations (data not shown).

**Figure 5:**
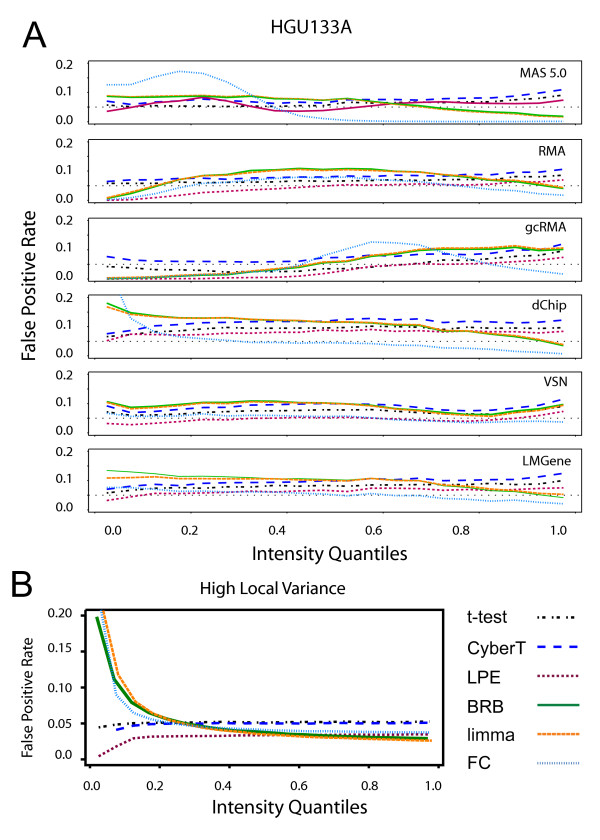
**False positive rates across concentration**. False positive rates across concentration using a *p *< 0.05 threshold. Genes are partitioned into 20 equal-sized bins ordered on expression intensity; for each bin the FPR is calculated by dividing the number of significant genes in the bin by the total number of genes in the bin. (A) HGU133A Latin Square data with "spiked-in" genes removed. The dashed line is the expected 0.05 FPR rate. (B) Simulated local variance model null hypothesis data with high variance (*n *= 3). Differential expression tests: FC: Fold Change, limma: limma moderated t-statistic, BRB: BRB t-statistic, CyberT: CyberT t-statistic, LPE: LPE z-statistic, t-stat: t-statistic. The limma and BRB t-statistic produce comparable results and consequently limma's orange line often conceals BRB's green line.

The effects of expression intensity on the FPR with the HGU133A Latin Square data varied across differential expression tests (Figure 5A). LPE showed a decreasing FPR at low concentrations, with the exception of the MAS 5.0 data. By contrast, the FPR for the t-statistic and CyberT t-statistic were more uniform across concentration.

FC showed an unstable FPR across intensity with all normalization methods. The FC FPR curve closely follows the smoothing curve of pooled variance versus intensity from Figures 1A and 1C for each normalization method. The FC FPR increases as the average variability at a particular intensity is high and decreases where the average variability is low. For example, with the HGU133A data normalized with dChip, there are generally many genes with high variance at low intensities and as the intensity increases the gene variances tend to decrease. The corresponding FPR follows a similar curve, with a high FPR for genes with low intensities and a low FPR for genes with high intensities. The FC method is unable to distinguish between random and biological differences in gene expression when the variability of the replicate measurements is high.

The CyberT t-statistic and limma moderated t-statistic also showed a similar, although weaker, pattern as FC in its FPR behaviour across concentration. This is due to the pooling of each gene-specific variance with a single prior error estimate. Genes with variances higher than the prior estimate have their variances reduced and genes with low variances have their variances increased. This causes the denominator of the t-statistic formula to decrease where variance is generally high, making it more likely that the null hypothesis is rejected. The opposite effect will happen when variance is relatively low. The two tests had a stable FPR with the VSN and LMGene normalized HGU133A data as the use of a glog transformation stabilized the variance more effectively across intensity.

For the simulated local variance model, FC, the BRB t-statistic and limma moderated t-statistic (Figure 5B) showed a similar tracking of the pooled variance smoothing curve of Figure 1A. Thus, the limma and BRB tests' superior pAUC performance (Figure 2A) and higher true positive rate at low concentrations (Figure 4B) for the local variance model is mitigated by its high FPR among low concentrations; for the local variance model, the t-statistic and the CyberT t-statistic alone yielded the expected 0.05 false positive rate across all concentrations. The LPE z-statistic yielded the lowest FPR across all concentrations.

## Conclusion

Consistent with the consensus among analysts that "borrowing strength" across genes for estimating differential expression random error is desirable [9,18,43], the three Empirical Bayes tests (BRB t-statistic, CyberT t-statistic, and limma moderated t-statistic) performed best. The t-statistic and the LPE z-statistic had reduced power in comparison. FC was comparable to the three Empirical Bayes methods with data of low variability (the Latin Square data with the exception of MAS 5.0 normalization and the cDNA liver3 versus liver5 data) but performed poorly with data characterized by high variability (the Latin Square data normalized with MAS 5.0 and the cDNA liver versus pooled data). This is in accordance with other studies that have shown the standard t-statistic to be suboptimal [2,18-20] and that FC is an inappropriate inferential test [9].

LPE introduced a bias that caused it to generate fewer false positives than expected. This bias appears to be due to a theoretical weakness and an incorrect assumption intrinsic to the test as originally proposed. Murie and Nadon [44] have shown that the adjustment to the LPE standard error, which depends on an asymptotic proof, is overly conservative with sample sizes smaller than 100. Moreover, empirical evidence suggests that the LPE's assumption of similar error variability for genes of similar expression intensity is incorrect, which leads the LPE test to overestimate p-values for low variability genes and to underestimate them for high variability genes. Recent modifications to the test have addressed this issue for microarray gene expression [45,46] and for mass spectrometry proteomics data [47]. Indeed, potential differences in high and low variability genes between our studies and that of Kooperberg et al. [15] may explain why they concluded that the LPE test was overly liberal in contrast to our current findings and those of Murie and Nadon that the test is overly conservative.

An important difference in our study between the CyberT test and the other two Empirical Bayes tests (BRB and limma), was that only the former maintained a stable false positive error rate with data that showed an unstable variance across intensity, such as the experimental and local normal simulated data. This suggests that these latter tests may also generate too many false positives among some biological data sets which, depending on pre-processing, often show high variance at low concentrations.

As designed in our simulations and has been argued generally, however, this variance/intensity relationship is an artifact of the inappropriate log transformation for low intensity expression values and the generalized log (glog) transformation is able to stabilize the error variance across the entire concentration range [30,48]. The glog transformation, as implemented with VSN and LMGene, lowers this potentially high FPR among low abundance transcripts in biological data sets while maintaining a relatively high TPR.

Background correction when applied to cDNA data can generate negative values which cannot be logged and requires that the negative data points be either discarded or set to a loggable value. Moreover, we found, as did Qin and Kerr [2], that using a log transformation after applying background correction in conjunction with loess normalization either reduced performance, as with the liver vs pool data, or had a mixed effect on performance, as with the liver vs liver data. We recommend the use of a glog transformation (LMGene) when applying background correction which can transform negative values and thus avoids the loss of information. The combination of glog and loess normalization produced comparable results for both cDNA data sets with and without background correction and were amongst the top performers of all normalization methods. An attractive alternative might be to use background correction methods which avoid negative values and stabilize the variance as a function of intensity as described in Ritchie et al. [49].

The performance of the statistical tests was most similar using the HGU133A data set. With the exception of MAS 5.0 with FC, all of the statistical tests found most of the "spiked-in" genes readily, illustrating the limitations of these empirical data sets for comparing performances of statistical test as they would be applied in experiments with biological variation. The HGU133A data set, which used technical replicates, is comparable to our simulated data with low variance, where in general the methods do not show large performance differences. The HGU95 data had higher variability than the HGU133A data and also showed greater performance differences between methods. Most biological experiments will be closer to the medium and the high variance simulated data sets where differences in performance are more apparent. The three Empirical Bayes methods in our study consistently performed well across normalization methods and simulated data designed to mirror the greater variability that is observed in most biological experiments. For this reason, we expect that our results apply beyond the narrow methodological confines of our study. Nonetheless, more definitive tests of the algorithms' merits will be made possible by future spike-in experiments which are anticipated to incorporate biological variability [50]. There is also a need for a similar comparative study among statistical tests which are suited to study designs that are more complex than the two-independent sample design in our study. Two of the approaches we evaluated (limma and BRB) and at least one other that we did not examine [17] have more general capabilities suited to this type of comparative study.

## Authors' contributions

CM conducted the computer simulations, performed the data analysis, participated in research design, and drafted the manuscript. OW and AL participated in discussion of the research, investigation of the statistical methods, and analysis of data. RN conceived of the study, supervised research design, interpretation of results, and drafting of the manuscript.

## Supplementary Material

Additional file 1 **MvA plots of cDNA data.** Click here for file

Additional file 2 **Source code availability**. Click here for file

Additional file 3 **Histograms of logged and unlogged variances for limma and BRB statistical tests.** Click here for file

Additional file 4 **pAUC scores using a 5% false positive rate.** Click here for file

Additional file 5 **Trellis plots of performance criteria for simulated data.** Click here for file

Additional file 6 **Total average rankings of statistical tests for pAUC and TPR.** Click here for file
