# Supplementary material for: Comparison of small n statistical tests of differential expression applied to microarrays
Source: BMC Bioinformatics. 2009 Feb 3;10:45. doi: 10.1186/1471-2105-10-45 (PMC2674054; doi:10.1186/1471-2105-10-45)
Supplement: Additional file 1 — MvA plots of cDNA data. [file 1471-2105-10-45-S1.pdf]

# Supplementary Materials: Comparison of small n statistical tests of differential expression applied to microarrays Additional File 1

Carl Murie, Owen Woody, Anna Y. Lee , Robert Nadon

January 27, 2009

## 1 MvA plots of cDNA data

The blue line in the MvA graphs shows the lowess fit of only the non-spike data points. The purple line in the MvA graph shows the lowess fit of both the spikes and non-spikes. In all cases the two lowess fits are virtually identical.

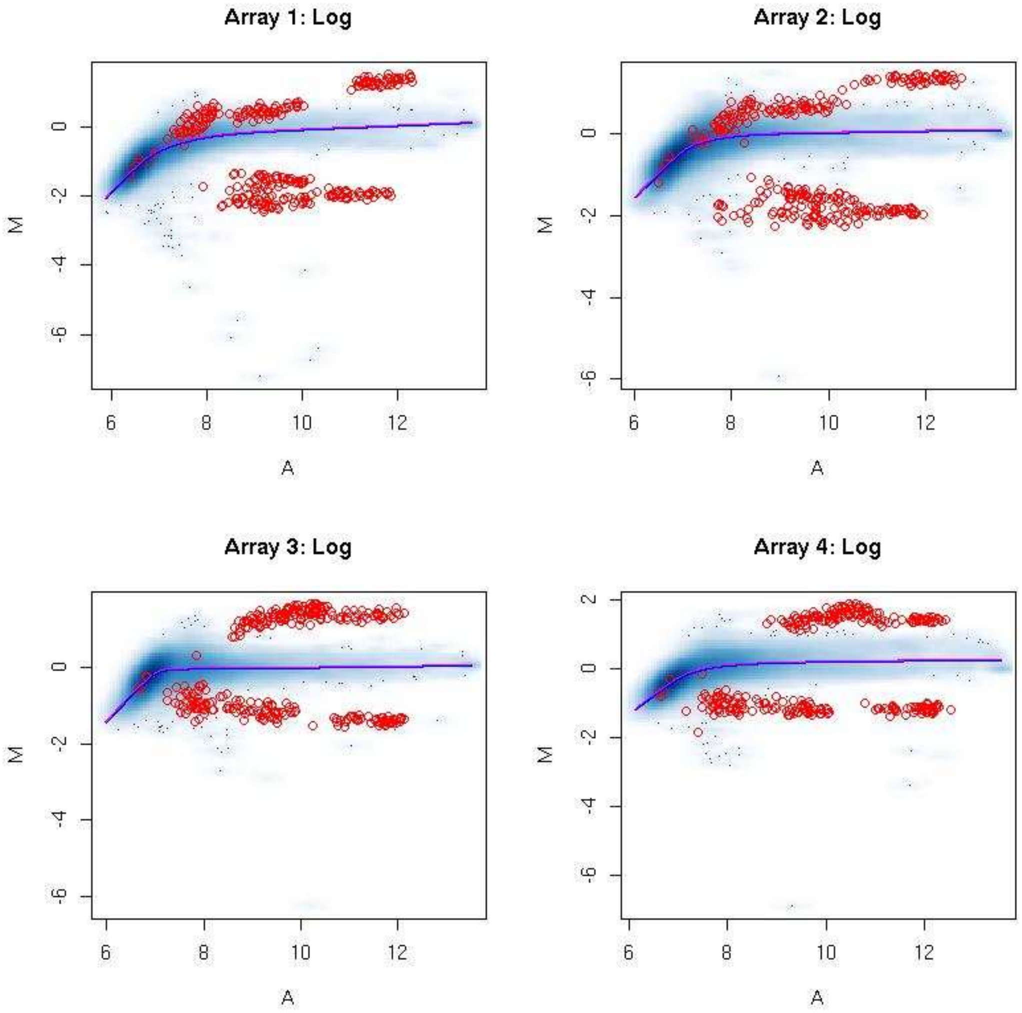


Figure 1: Liver3v5 MvA plot with log data. Red points are spikes and blue points are non-spikes.

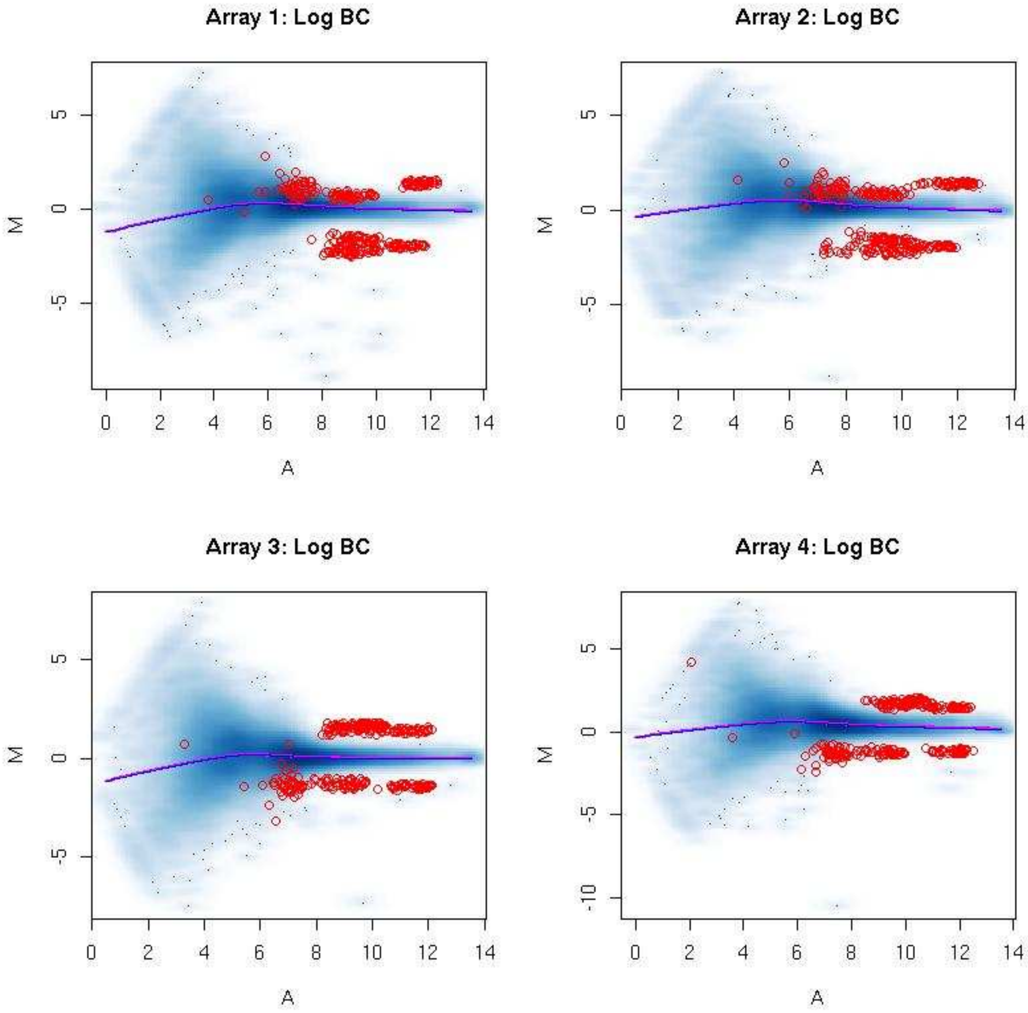


Figure 2: Liver3v5 MvA plot with log data after background correction. Red points are spikes and blue points are non-spikes.

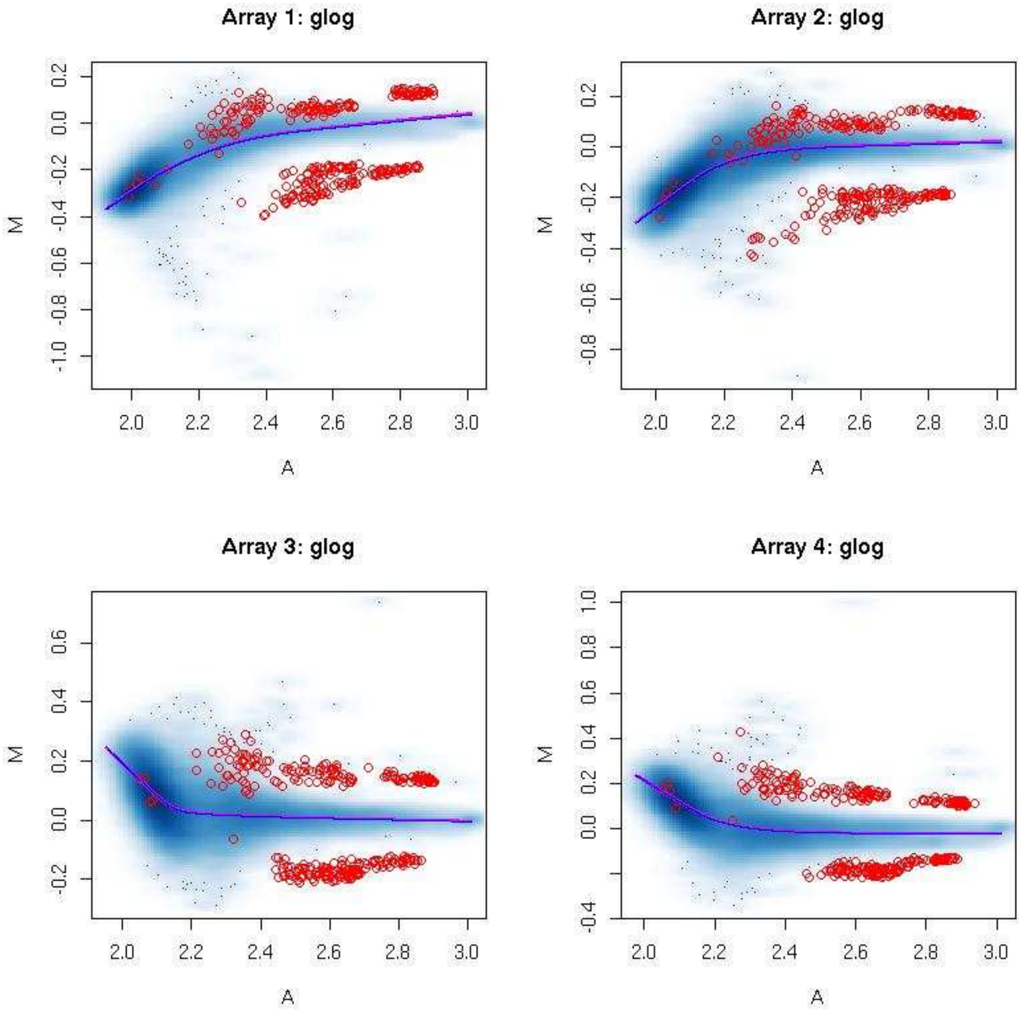


Figure 3: Liver3v5 MvA plot with glog data. Red points are spikes and blue points are non-spikes.

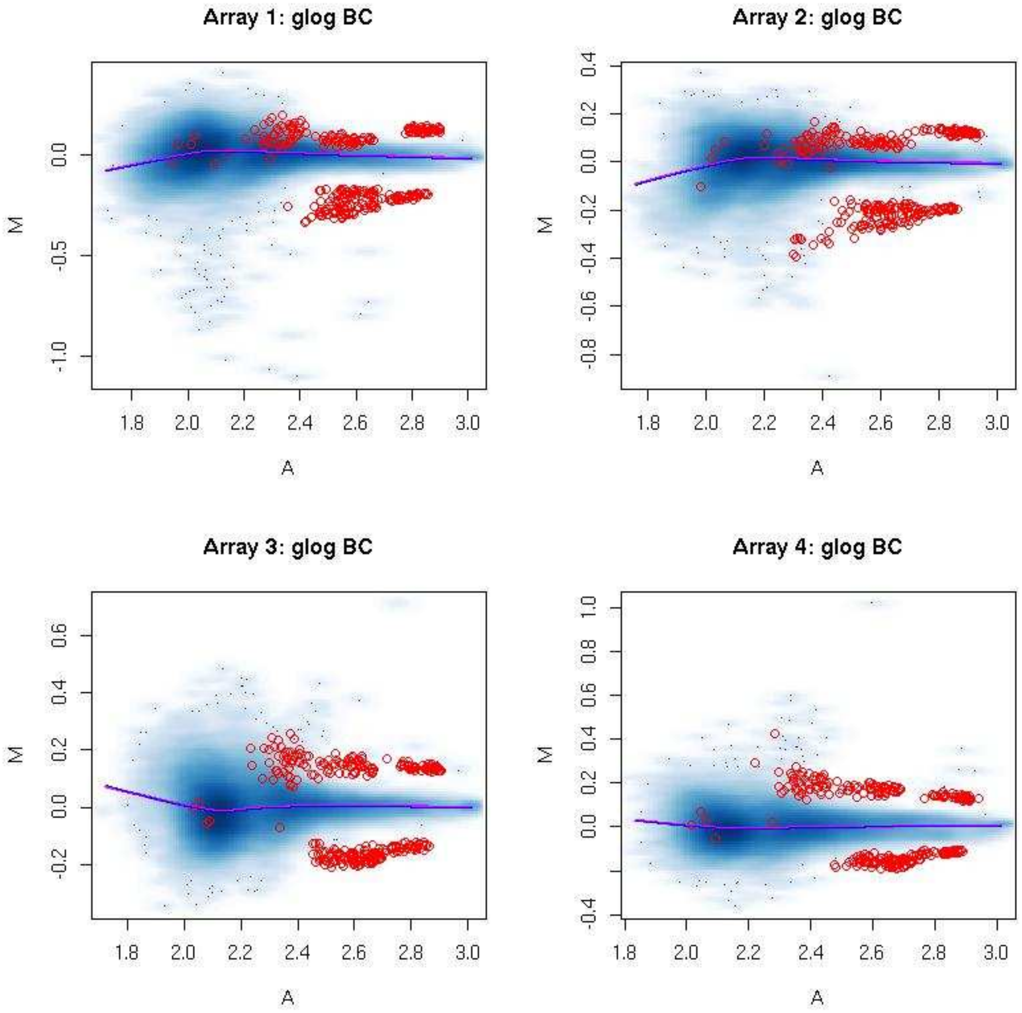


Figure 4: Liver3v5 MvA plot with glog data after background correction. Red points are spikes and blue points are non-spikes.

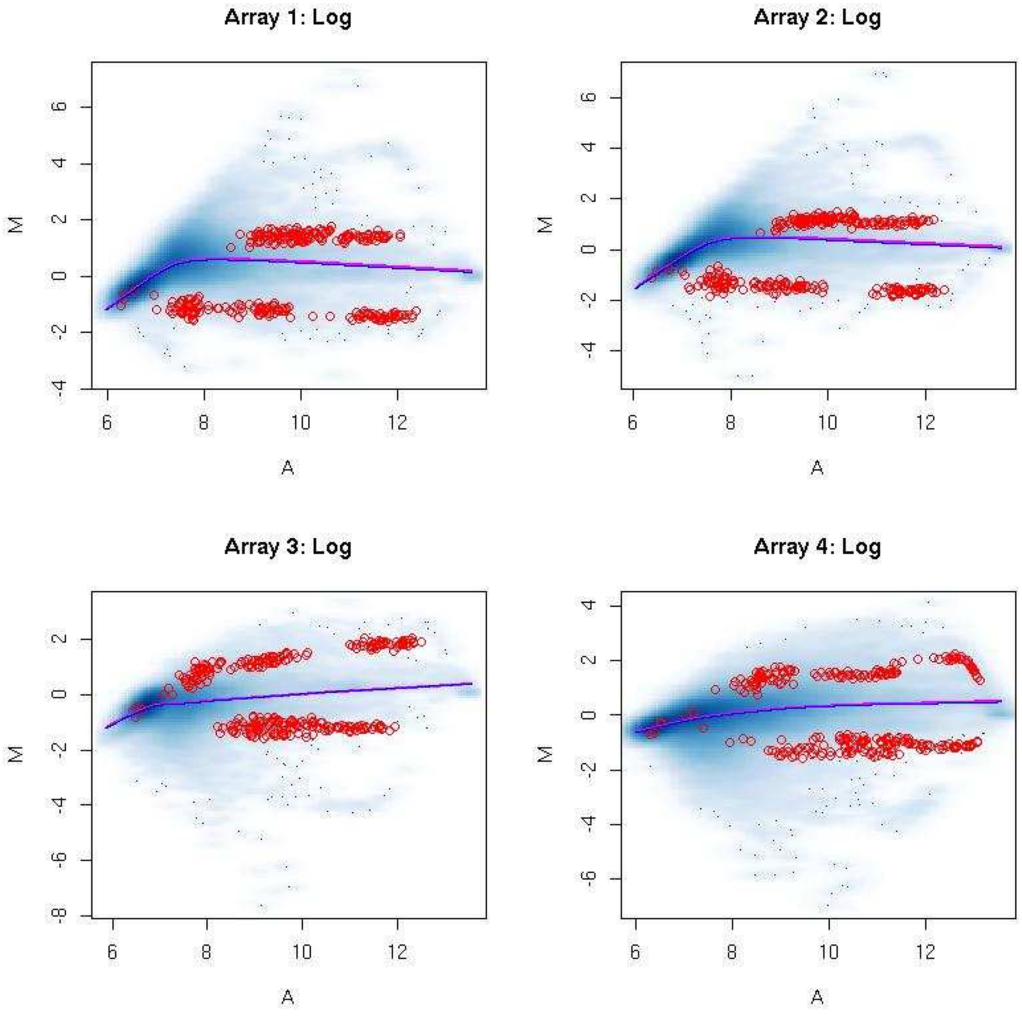


Figure 5: Liver pooled MvA plot with log dat. Red points are spikes and blue points are non-spikes.a

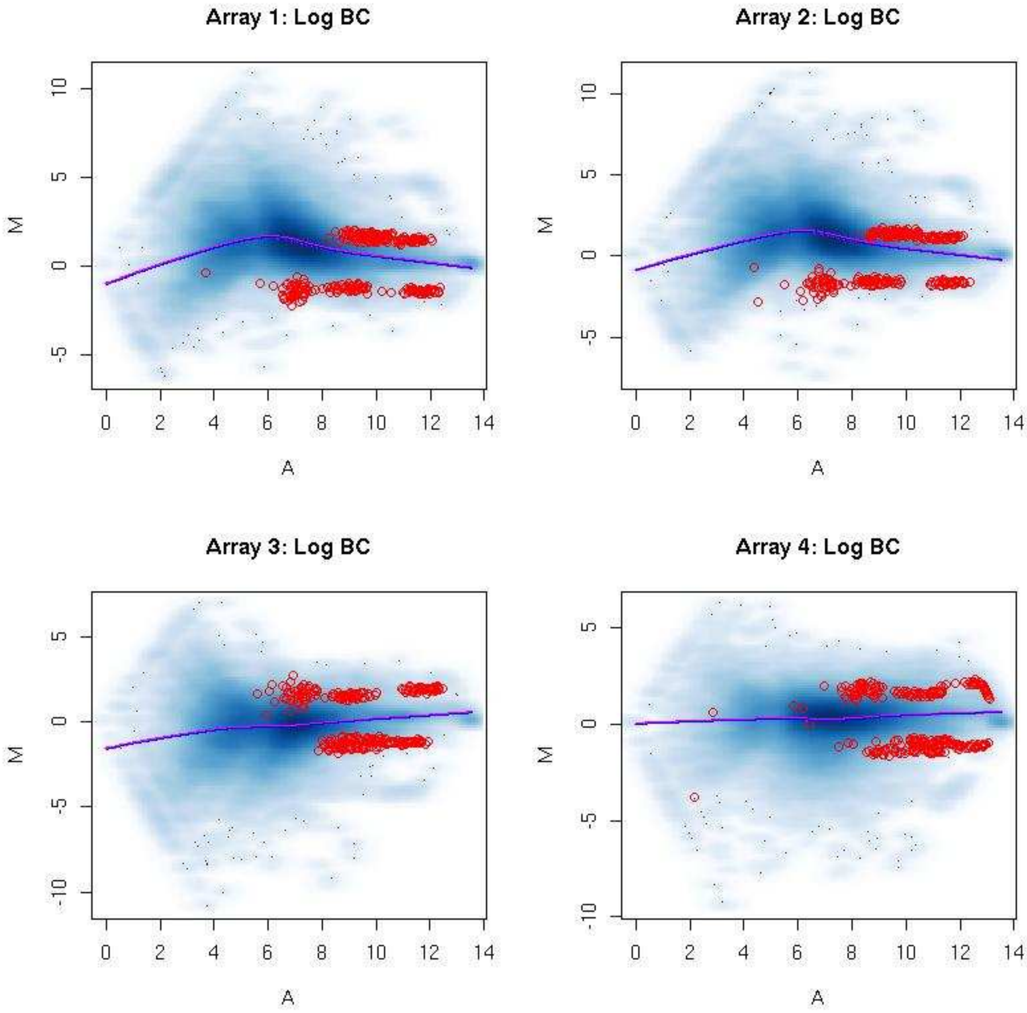


Figure 6: Liver pooled MvA plot with log data after background correction. Red points are spikes and blue points are non-spikes.

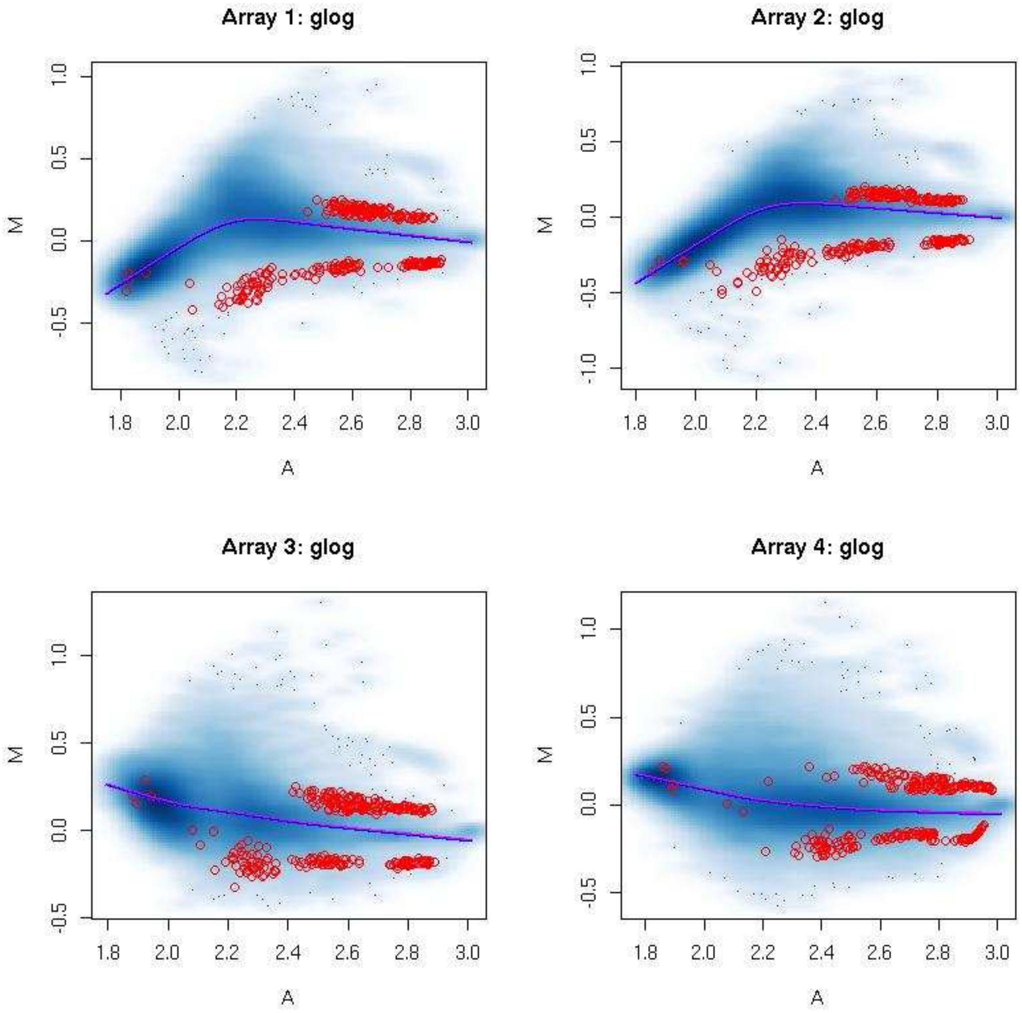


Figure 7: Liver pooled MvA plot with glog data. Red points are spikes and blue points are non-spikes.

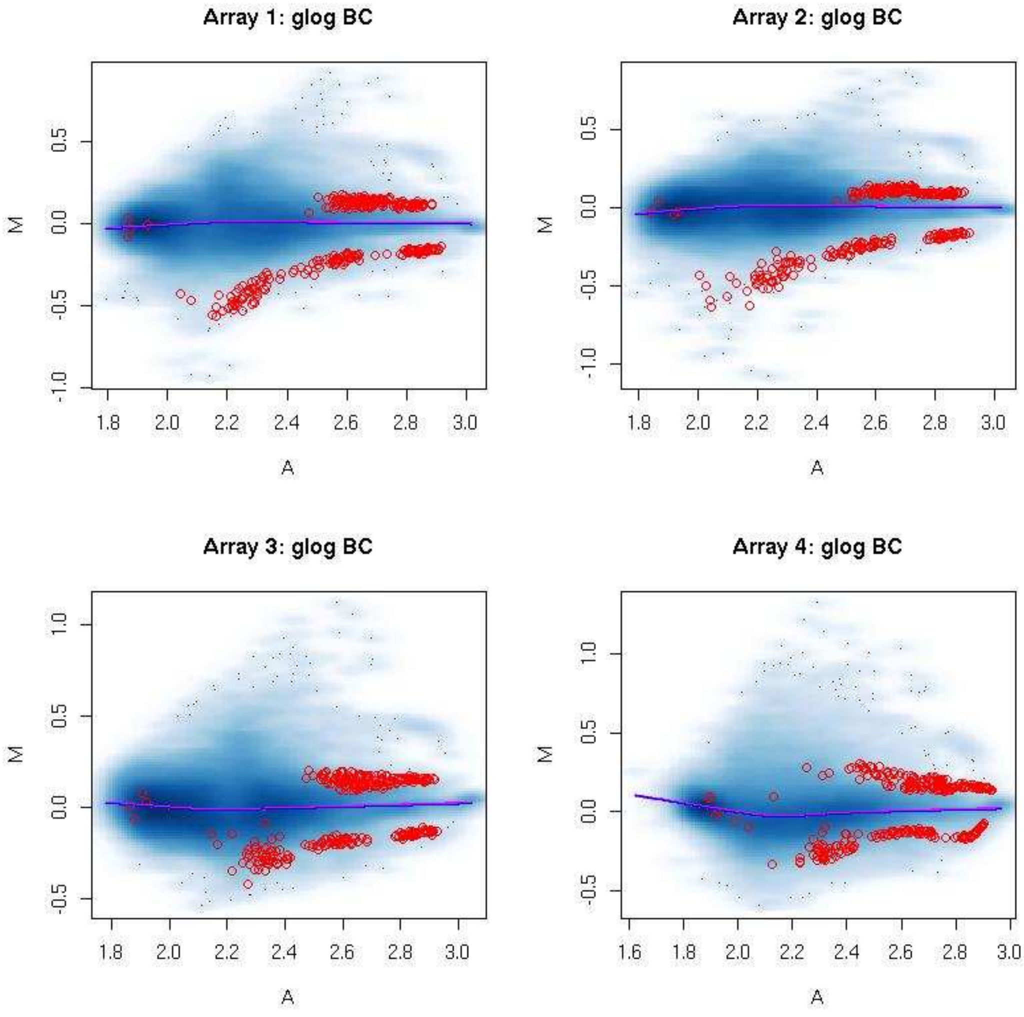


Figure 8: Liver pooled MvA plot with glog data after background correction. Red points are spikes and blue points are non-spikes.
