# Supplementary material for: Comparison of small n statistical tests of differential expression applied to microarrays
Source: BMC Bioinformatics. 2009 Feb 3;10:45. doi: 10.1186/1471-2105-10-45 (PMC2674054; doi:10.1186/1471-2105-10-45)
Supplement: Additional file 2 — Source code availability. [file 1471-2105-10-45-S2.pdf]

Supplementary Materials: Comparison of small n statistical tests of differential expression applied to microarrays Additional File 2

Carl Murie, Owen Woody, Anna Y. Lee , Robert Nadon

January 27, 2009

1 Source Code Availability

- R statistical language - version 2.5.1
  (http://www.r-project.org)
- MAS 5.0:
  bioconductor affy library - version 1.13
  (http://www.bioconductor.org)
- RMA:
  bioconductor affy library - version 1.13
  (http://www.bioconductor.org)
- gcRMA:
  bioconductor affy library - version 1.13
  (http://www.bioconductor.org)
- dChip:
  bioconductor affy library - version 1.13
  (http://www.bioconductor.org)
- LPE z statistic:
  bioconductor LPE library - version 1.10
  (http://www.bioconductor.org)

- LMGene:
  bioconductor LMGene library - version 1.6
  (http://www.bioconductor.org)
- limma:
  bioconductor limma library - 2.10.5
  (http://www.bioconductor.org)
- BRB t-statistic:
  Wright and Simon -BRB Array Tools
  (http://linus.nci.nih.gov/BRB-ArrayTools.html)
- CyberT t-statistic:
  (http://cybert.microarray.ics.uci.edu/)
