# Supplementary material for: Comparison of small n statistical tests of differential expression applied to microarrays
Source: BMC Bioinformatics. 2009 Feb 3;10:45. doi: 10.1186/1471-2105-10-45 (PMC2674054; doi:10.1186/1471-2105-10-45)
Supplement: Additional file 3 — Histograms of logged and unlogged variances for limma and BRB statistical tests. [file 1471-2105-10-45-S3.pdf]

Supplementary Materials: Comparison of small n statistical tests of differential expression applied to microarrays Additional File 3

Carl Murie, Owen Woody, Anna Y. Lee , Robert Nadon

January 27, 2009

1 Histograms of logged and unlogged variances for limma and BRB statistical tests

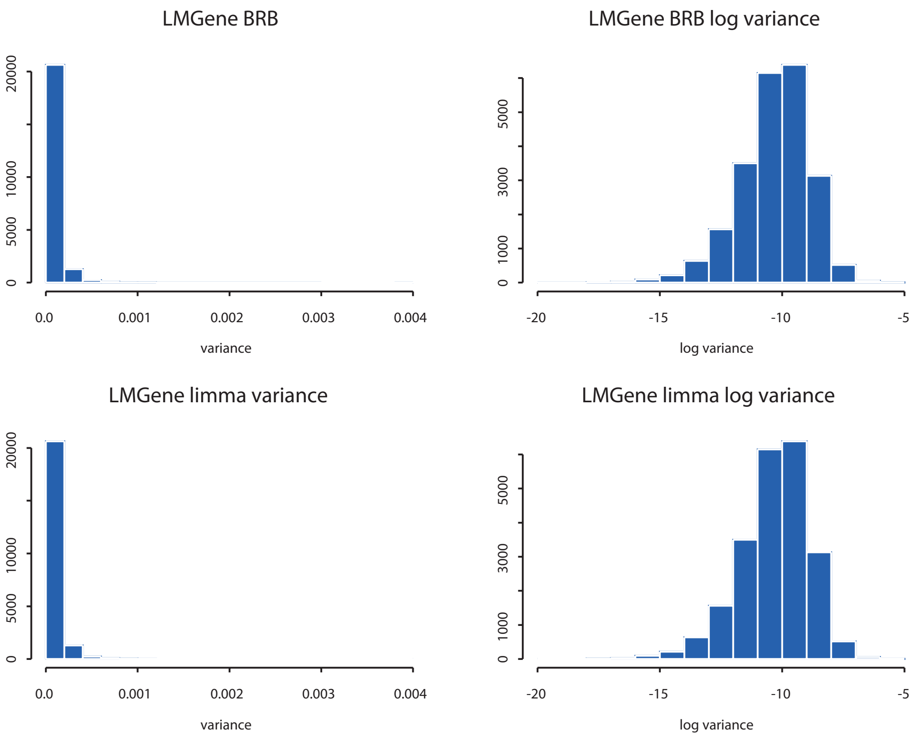

Figure 1: Histograms of posterior variances, logged and unlogged, for BRB and limma statistical tests using lmGene normalized data.

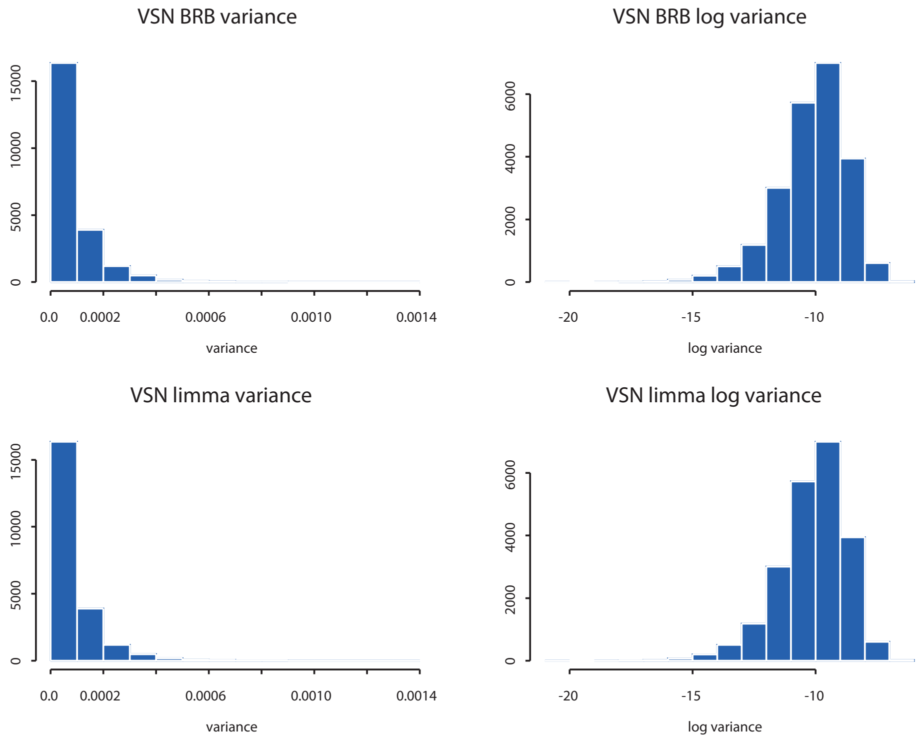

Figure 2: Histograms of posterior variances, logged and unlogged, for BRB and limma statistical tests using VSN normalized data.
