# Supplementary material for: Comparison of small n statistical tests of differential expression applied to microarrays
Source: BMC Bioinformatics. 2009 Feb 3;10:45. doi: 10.1186/1471-2105-10-45 (PMC2674054; doi:10.1186/1471-2105-10-45)
Supplement: Additional file 4 — pAUC scores using a 5% false positive rate. [file 1471-2105-10-45-S4.pdf]

Supplementary Materials: Comparison of small n statistical tests of differential expression applied to microarrays
Additional File 4

Carl Murie, Owen Woody, Anna Y. Lee , Robert Nadon

January 27, 2009

1 pAUC scores: Calculated using a 5% false positive rate

Table 1: Rankings from best to worst (1 to 5) of average pAUC scores (using a threshold of five percent of the false positives) of the six tests of differential expression. The average pAUC for each score is in parentheses. Differential tests are t-stat: t-statistic, LPE: LPE z-statistic, CyberT: CyberT t-statistic, limma: limma moderated t-statistic, BRB: BRB t-statistic, FC: Fold Change. The lmGene package was used to apply the Loess normalization and glog transformation to the cDNA data. BC indicates that background correction was applied to the data before normalization.

		**t-stat**	**CyberT**	**LPE**	**BRB**	**limma**	**FC**	median pAUC
HGU133A	MAS 5.0	5 (.67)	1 (.72)	4 (.68)	2.5 (.69)	2.5 (.69)	6 (.18)	.69
	RMA	6 (.73)	3.5 (.84)	3.5 (.84)	3.5 (.84)	3.5 (.84)	1 (.86)	.84
	gcRMA	6 (.75)	2.5 (.84)	2.5 (.84)	4 (.79)	5 (.78)	1 (.86)	.82
	dChip	4.5 (.78)	3 (.80)	6 (.77)	1.5 (.81)	1.5 (.81)	4.5 (.78)	.79
	LMGene	6 (.73)	3.5 (.84)	3.5 (.84)	1.5 (.86)	5 (.82)	1.5 (.84)	.84
	VSN	6 (.73)	4 (.83)	4 (.83)	1.5 (.85)	4 (.83)	1.5 (.85)	.83
	avg. rank	5.6	2.9	3.9	2.4	3.6	2.6	
HGU95	MAS 5.0	3 (.61)	1 (.73)	2 (.64)	4.5 (.46)	4.5 (.46)	6 (.10)	.54
	RMA	6 (.72)	1.5 (.87)	1.5 (.87)	3.5 (.85)	3.5 (.85)	5 (.84)	.85
	gcRMA	6 (.87)	1 (.95)	3 (.93)	3 (.93)	3 (.93)	5 (.92)	.93
	dChip	6 (.76)	3 (.84)	4 (.82)	1.5 (.86)	1.5 (.86)	5 (.81)	.83
	LMGene	6 (.76)	2 (.87)	1 (.89)	4 (.86)	4 (.86)	4 (.86)	.86
	VSN	6 (.61)	5 (.79)	3.5 (.82)	1.5 (.83)	3.5 (.82)	1.5 (.83)	.82
	avg. rank	5.5	2.3	2.5	3.0	3.3	4.4	
cDNA liver vs liver	Loess	5.5 (.97)	2.5 (.98)	2.5 (.98)	2.5 (.98)	2.5 (.98)	5.5 (.97)	.98
	Loess(BC)	5 (.96)	1 (.99)	2 (.98)	3.5 (.97)	3.5 (.97)	6 (.66)	.97
	glog Loess	6 (.94)	3 (.98)	3 (.98)	3 (.98)	3 (.98)	3 (.98)	.98
	glog Loess(BC)	6 (.93)	1.5 (.98)	1.5 (.98)	3.5 (.97)	3.5 (.97)	5 (.94)	.97
	VSN	6 (.95)	2.5 (.98)	2.5 (.98)	2.5 (.98)	2.5 (.98)	5(.97)	.98
	avg. rank	5.7	2.1	2.3	3.0	3.0	4.9	
cDNA liver vs pool	Loess	6 (.07)	5 (.08)	1 (.26)	3.5 (.09)	3.5 (.09)	2 (.17)	.09
	Loess(BC)	2(.03)	5 (.02)	5 (.02)	2 (.03)	2 (.03)	5 (.02)	.03
	glog Loess	1 (.16))	4 (.13)	2 (.14)	4 (.13)	4 (.13)	6 (.08)	.13
	glog Loess(BC)	1.5 (.15)	1.5 (.15)	3 (.14)	4.5 (.09)	4.5 (.09)	6 (.06)	.12
	VSN	3 (.10)	4 (.09)	5.5 (.05)	1.5 (.11)	1.5 (.11)	5.5 (.05)	.10
	avg. rank	2.7	3.9	3.3	3.1	3.1	4.9	
Simulated	Common	6 (.32)	3 (.54)	5 (.46)	3 (.54)	1 (.55)	3 (.54)	.54
	Local	6 (.16)	4 (.28)	5 (.21)	2 (.61)	1 (.62)	3 (.31)	.30
	Inverse Gamma	5 (.40)	3 (.53)	6 (.39)	1.5 (.54)	1.5 (.54)	4 (.48)	.50
	avg. rank	5.7	3.3	5.3	2.2	1.1	3.3	
	total avg. rank	5.0	2.9	3.5	2.7	2.8	4.0	
