# Supplementary material for: Comparison of small n statistical tests of differential expression applied to microarrays
Source: BMC Bioinformatics. 2009 Feb 3;10:45. doi: 10.1186/1471-2105-10-45 (PMC2674054; doi:10.1186/1471-2105-10-45)
Supplement: Additional file 5 — Trellis plots of performance criteria for simulated data. [file 1471-2105-10-45-S5.pdf]

Supplementary Materials: Comparison of small n statistical tests of differential expression applied to microarrays Additional File 5

Carl Murie, Owen Woody, Anna Y. Lee , Robert Nadon

January 28, 2009

1 Simulated Data: Trellis Plots Of Performance Criteria

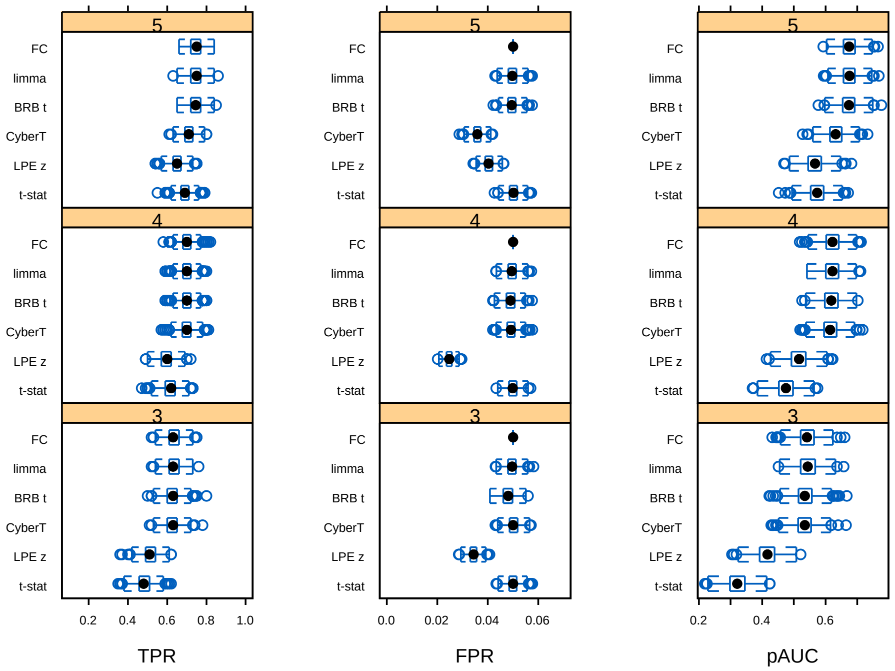

Figure 1: Simulated Data Common Variance Model - High Variance: Performance statistics trellis plot.

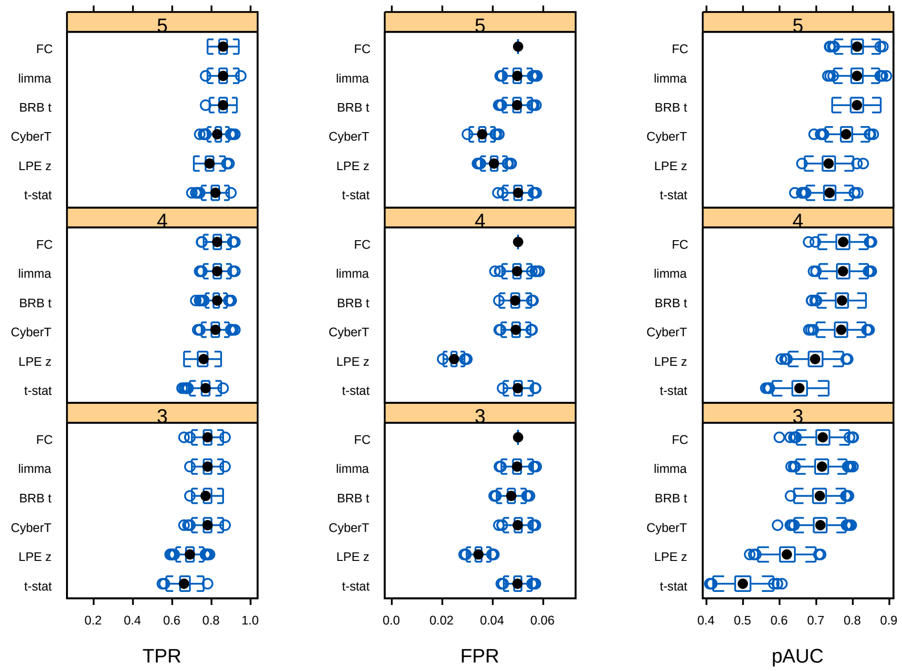

Figure 2: Simulated Data Common Variance Model - Medium Variance: Performance statistics trellis plot.

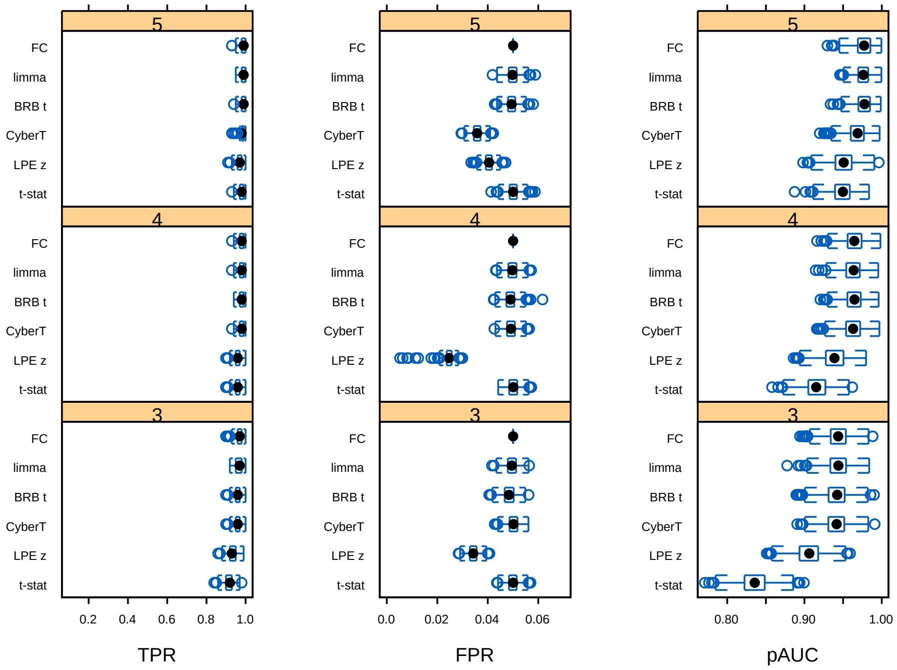

Figure 3: Simulated Data Common Variance Model - Low Variance: Performance statistics trellis plot.

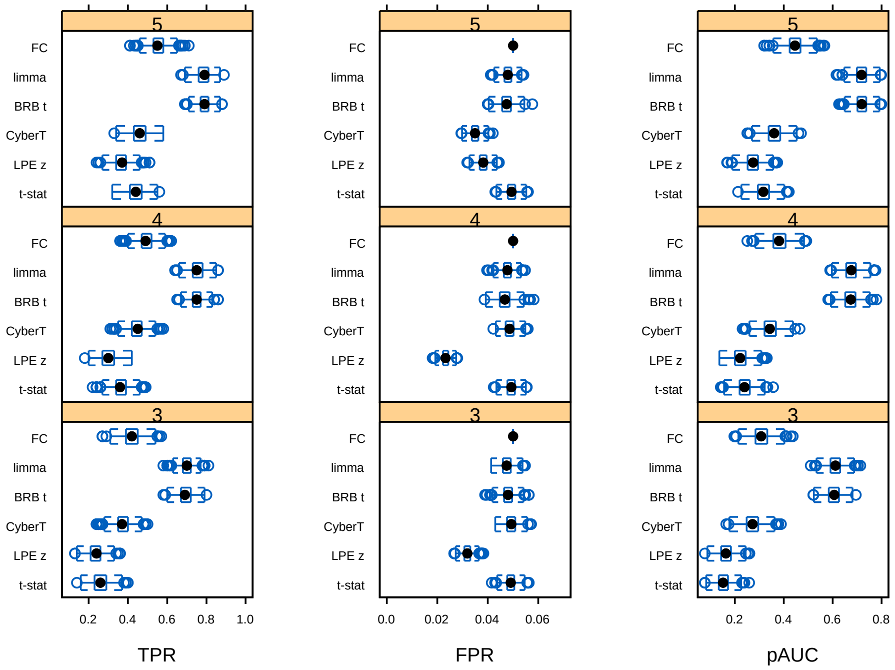

Figure 4: Simulated Data Local Variance Model - High Variance: Performance statistics trellis plot.

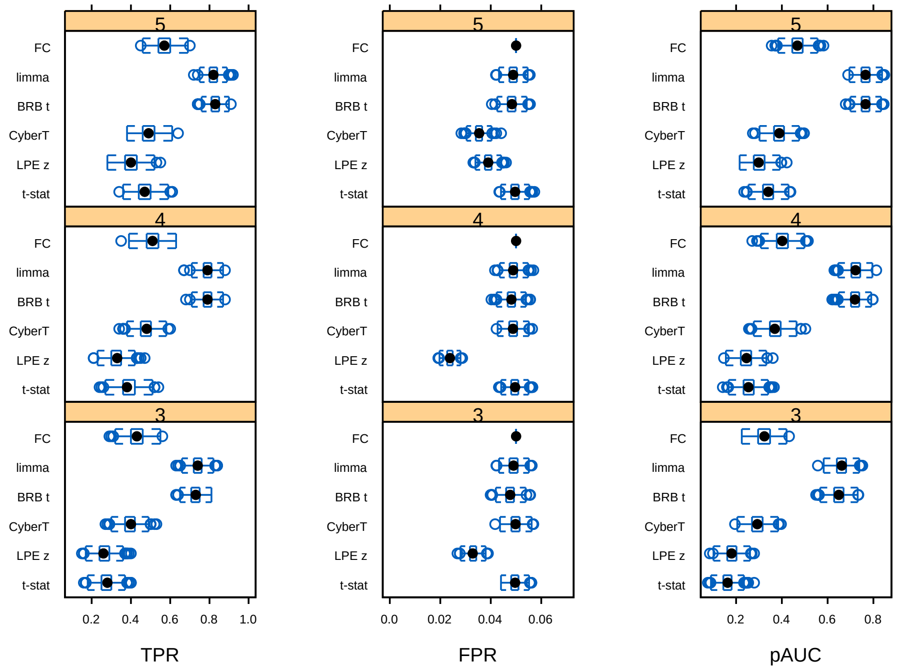

Figure 5: Simulated Data Local Variance Model - Medium Variance: Performance statistics trellis plot.

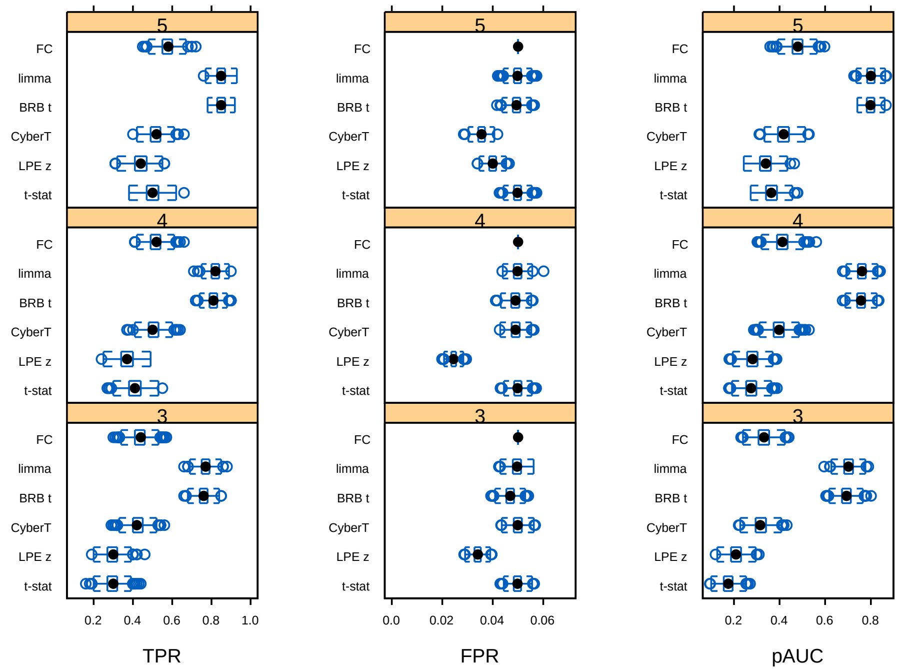

Figure 6: Simulated Data Local Variance Model - Low Variance: Performance statistics trellis plot.

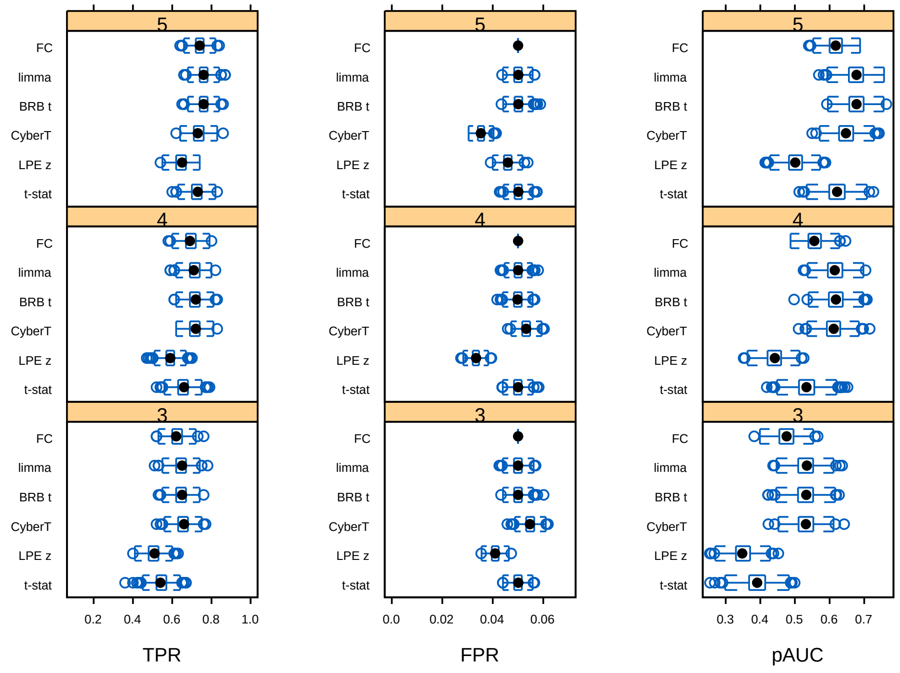

Figure 7: Simulated Data Inverse Gamma Variance Model - High Variance: Performance statistics trellis plot.

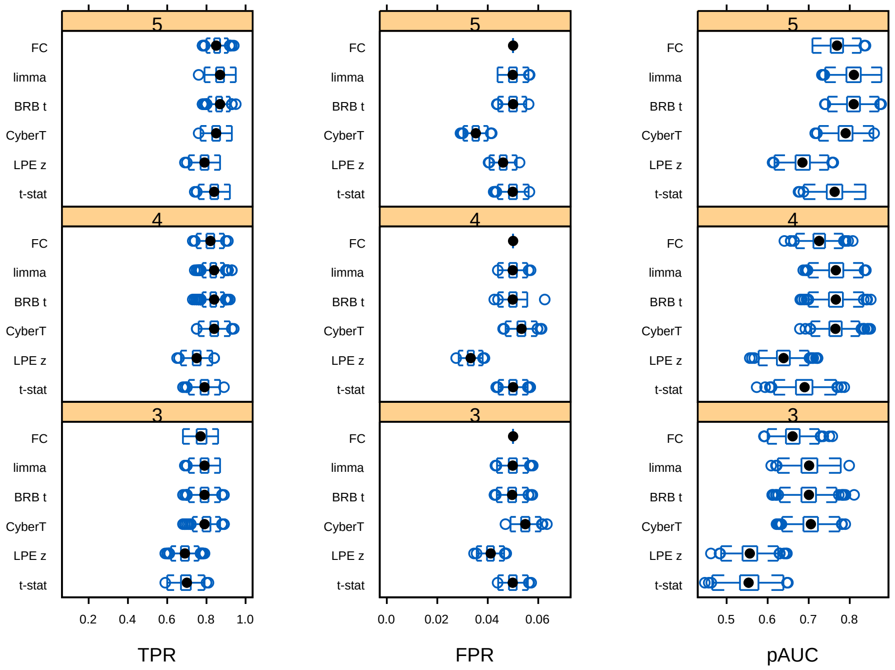

Figure 8: Simulated Data Inverse Gamma Variance Model - Medium Variance: Performance statistics trellis plot.

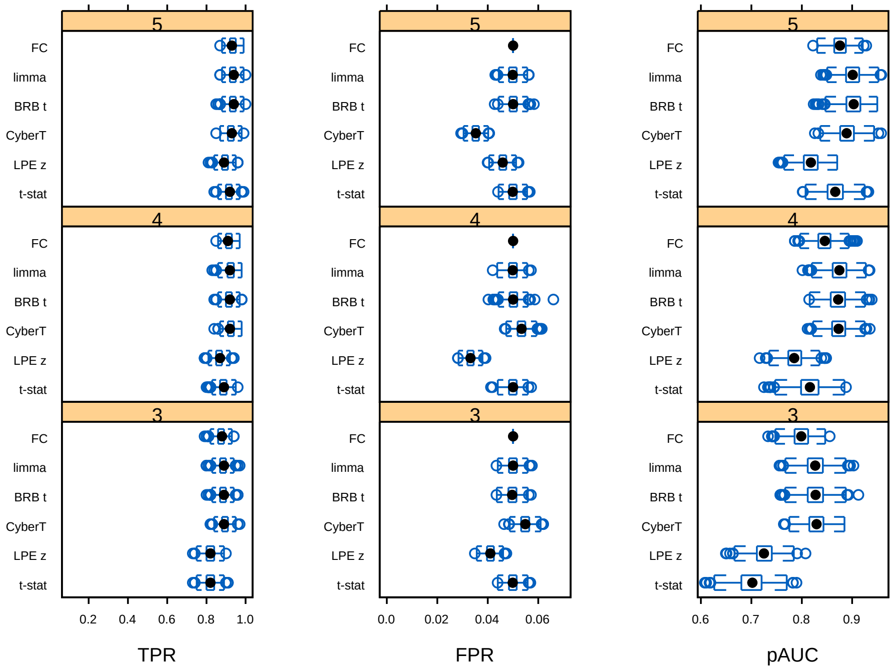

Figure 9: Simulated Data Inverse Gamma Variance Model - Low Variance: Performance statistics trellis plot.
