# Supplementary material for: Comparison of small n statistical tests of differential expression applied to microarrays
Source: BMC Bioinformatics. 2009 Feb 3;10:45. doi: 10.1186/1471-2105-10-45 (PMC2674054; doi:10.1186/1471-2105-10-45)
Supplement: Additional file 6 — Total average rankings of statistical tests for pAUC and TPR. [file 1471-2105-10-45-S6.pdf]

Supplementary Materials: Comparison of small n statistical tests of differential expression applied to microarrays
Additional File 6

Carl Murie, Owen Woody, Anna Y. Lee , Robert Nadon

January 28, 2009

1 Total average rankings of statistical tests for pAUC and TPR

Table 1: Average rankings of the 6 statistical tests for the pAUC using a 5% pvalue cutoff (Table 2 of the manuscript), the pAUC using a 5% false positive rate cutoff (Additional file 4), and the true positive rate (TPR) (Table 3 of the manuscript).

	**t-stat**	**CyberT**	**LPE**	**BRB**	**limma**	**FC**
pAUC (p value)	5.0	2.2	5.0	2.6	2.3	3.7
pAUC (FPR)	5.0	2.9	3.5	2.7	2.8	4.0
TPR	5.0	2.3	5.1	2.5	2.9	3.1
